# Cellular and Subcellular Contact Guidance on Microfabricated Substrates

**DOI:** 10.3389/fbioe.2020.551505

**Published:** 2020-10-22

**Authors:** Claire Leclech, Catherine Villard

**Affiliations:** ^1^Hydrodynamics Laboratory, CNRS UMR 7646, Ecole Polytechnique, Palaiseau, France; ^2^Physico-Chimie Curie, CNRS UMR 168, Université PSL, Sorbonne Université, Paris, France

**Keywords:** contact guidance, microfabricated substrates, topography, cellular responses, topography sensing, intracellular mechanisms

## Abstract

Topography of the extracellular environment is now recognized as a major biophysical regulator of cell behavior and function. The study of the influence of patterned substrates on cells, named contact guidance, has greatly benefited from the development of micro and nano-fabrication techniques, allowing the emergence of increasingly diverse and elaborate engineered platforms. The purpose of this review is to provide a comprehensive view of the process of contact guidance from cellular to subcellular scales. We first classify and illustrate the large diversity of topographies reported in the literature by focusing on generic cellular responses to diverse topographical cues. Subsequently, and in a complementary fashion, we adopt the opposite approach and highlight cell type-specific responses to classically used topographies (arrays of pillars or grooves). Finally, we discuss recent advances on the key subcellular and molecular players involved in topographical sensing. Throughout the review, we focus particularly on neuronal cells, whose unique morphology and behavior have inspired a large body of studies in the field of topographical sensing and revealed fascinating cellular mechanisms. We conclude by using the current understanding of the cell-topography interactions at different scales as a springboard for identifying future challenges in the field of contact guidance.

## Introduction

Throughout the whole life of multicellular organisms, cells live, and achieve their functions embedded in a highly complex and sometimes evolving 3D environment. The influence of external cues on cell function and behavior has long been envisioned mainly from a biochemical point of view (e.g., via the action of small chemicals, morphogens, hormones, growth factors, etc.). However, it is now clear that the physical properties of the extracellular environment, stiffness or topography for instance, play a crucial role in modulating cell morphology and major cell functions such as proliferation, differentiation, polarization, and migration.

The topography of the extracellular environment can be defined as the size, shape, and organization of structural elements present around cells. *In vivo*, most cells are surrounded by the extracellular matrix (ECM). The ECM of different tissues such as blood vessel, bone, cartilage, or nerve are composed of micro and nanoscale topographic features with which cells can interact. ECM structures can be composed of randomly organized elements like in basement membranes of blood vessels (Liliensiek et al., 2009) or in some ligaments or cartilages (Debski et al., 2003; Bi et al., 2005). In contrast, highly aligned ECM environments can be found, e.g., the cardiac interstitium or the corneal stroma, both composed of highly organized collagen fibers (Borg et al., 1996; Aghamohammadzadeh et al., 2004). Cells also interact with neighboring cells, whose shape can act as guiding tracks for migration, e.g., the long polarized basal process of radial glial cells for newborn cortical neurons (Paridaen and Huttner, 2014). Cells themselves can therefore create complex topographical environments at the micron scale affecting other cells. More generally, the short-scale dimensions and long-scale organization of the topographical cues are two important parameters to consider in the definition of the cell microenvironment topography.

The topography of the environment is thus a prominent element of cell physiology. It can also play a crucial role in pathological conditions such as cancer, where the tumor microenvironment can greatly influence cancerous cell behavior and dissemination. The remodeling of the stroma organization (e.g., the formation of bundles of aligned collagen fibers), by guiding cancer cell migration, has for instance been reported to be one of the key aspects of tumor invasion (Clark and Vignjevic, 2015).

The influence of the topography on cellular behaviors is referred to as contact guidance. As early as 1912, Ross G. Harrison mentioned not only the importance of a “solid support” for the movement of chick embryo cells in culture, but also noted that “when the latter has a specific linear arrangement (…) it has an action in influencing the direction of the movement, as well as upon the form and arrangement of the cells” (Harrison, 1912). Later, Paul Weiss reported another evidence of contact guidance *in vitro*. In particular, he noted that axons line up along parallel grooves formed by the “brushing” of coagulated blood onto mica coverslips (Weiss, 1934). Since then, contact guidance phenomenon has been described for many cell types using a wide range of structured substrates, either mimicking the structure and length scale of native *in vivo* topographies or offering challenging, artificial conditions to reveal hidden cellular properties (Tomba and Villard, 2015).

This burst of *in vitro* studies was supported by the emergence, from the 1990s, of micro and nano-fabrication techniques, and their dissemination in the field of cell biology. The great variety of materials and techniques used to create micro- and nanofabricated substrates, as well as the almost infinite possibilities of pattern designs results now in a large and diverse body of literature on the subject. Although we will not focus on the fabrication techniques available [on this subject see for instance (Norman and Desai, 2006)], it appeared essential to us in this context to provide a reference grid of the diversity of the reported observations.

The purpose of this review is thus, on the basis of a selection of the most salient results of the literature, to examine and link cell response to topography at different scales (cellular and subcellular). Our approach will be based on two complementary points of view, one considering cells for their generic properties and the other focusing on cellular specificities. The aim of this review is to provide an extensive report and overview of the field of contact guidance, linking the early descriptive studies with the most recent works and challenges in the field.

In a first and introductory section, we will classify in a limited number of categories the extensive range of topographies reported in the literature, highlighting the generic cell responses to each of them. We will mainly focus on cell morphology and, when relevant, cell migratory behavior. Conversely, we will consider in the second part of this review cell-type specific responses to selected categories of topography. Considering the unique branched and elongated morphology of neurons, we will in particular devote an entire subsection to the fascinating responses of these cells to topographical cues. In the two last parts of this review, we will dive into the subcellular and molecular scales of contact guidance. The third section will focus on topography sensing by exploratory subcellular structures such as filopodia or growth cones, before considering smaller structures, i.e., focal adhesions (FAs). We will review then in a last section the latest results and challenges regarding the molecular players involved in topography sensing. Finally, we will highlight the remaining open questions and challenges for the future in the conclusion of this review.

Throughout this review, we will focus on the cellular responses (i.e., morphology, migration) of isolated mammalian cells cultured on open 2D-substrates. Cell behavior in 3D environments or collective behaviors will not be treated here. Although we will mention some results on stem cells and topography-induced stem cell differentiation, this review is also not dedicated to this topic *per se*. The reader can find further and specific information on this rapidly expanding field in dedicated reviews (Chen et al., 2014; Metavarayuth et al., 2016; Simitzi et al., 2018; Huang et al., 2019; Xue et al., 2020).

## Generic Cellular Responses to a Large Repertoire of Microfabricated Topographies

**FIGURE 1:**
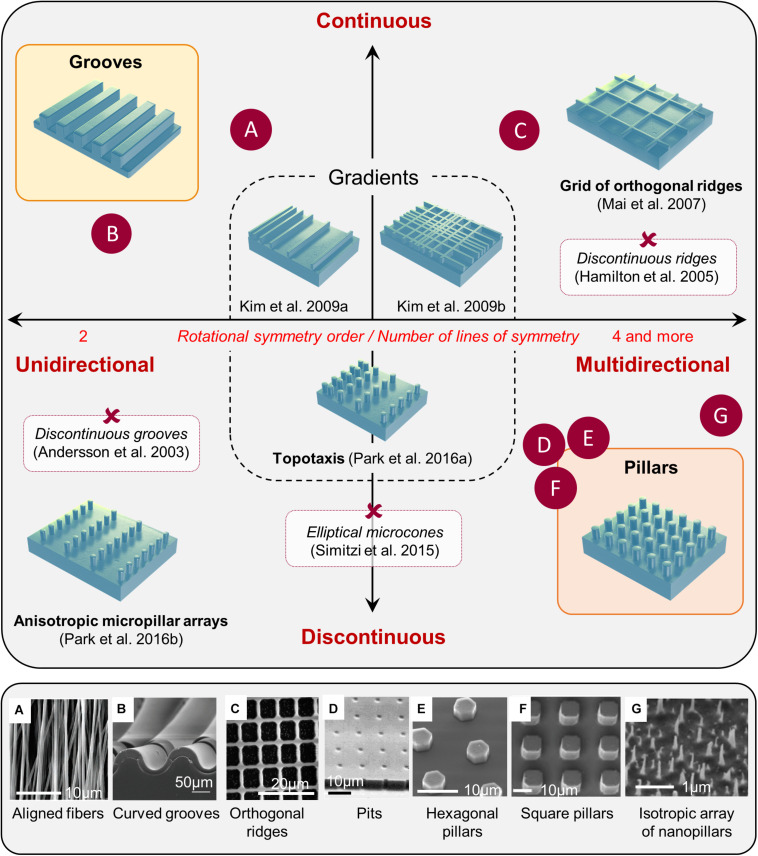
Classification of microstructured substrates. Most microstructured substrates found in the literature can be classified according to its rotational symmetry order (unidirectional toward multidirectional), and the continuous or discontinuous nature of the topographical cues. Examples of various substrates mentioned in the review are positioned in the classification (red crosses), and some examples are illustrated in the bottom panel (letters): reproduced with permission from **(A)** Adapted with permission from Patel et al. (2007). Copyright 2007 Americal Chemical Society. **(B)** Tomba et al. (2019); **(C)** Kim et al. (2009b); **(D)** Tan et al. (2000); **(E)** Micholt et al. (2013); **(F)** Leclech et al. (2019). **(G)** Adapted with permission from Patel et al. (2007). Copyright 2007 Americal Chemical Society.

It is now widely accepted that the topography of the cellular environment can greatly affect cells, ranging from basic cellular features, such as cell adhesion, morphology, or orientation to different cell functions such as cell proliferation, differentiation, polarization, or migration (see for reviews Ravichandran et al., 2009; Kim et al., 2012). Although we will not detail these effects here, it is still interesting to mention that substrate topography can be positively used in different *in vitro* cellular manipulations, decreasing cell stress (Puschmann et al., 2013) and increasing transfection efficiency (Adler et al., 2011), cell reprogramming (Yoo et al., 2015), or epigenetic state (Downing et al., 2013). A great variety of artificial microstructured substrates have been developed to study *in vitro* in a highly controlled manner the phenomenon of contact guidance (Figure 1). These different microfabricated topographies are classically separated into two main categories: unidirectional and multidirectional. Unidirectional topographies provide a continuous cue along a single axis and include the large categories of grooves topographies. Arrays of pillars or pits offer in contrast discontinuous cues in more than one direction. They have, often improperly, being gathered under the name of “isotropic” while they can mostly be described as multiple rotational symmetry (i.e., multidirectional) topographies. Purely isotropic environments (i.e., whose long-range order does not obey to any rotational axis or plane of symmetry, see Figure 1G) are more rarely used in the literature for mammalian cells (see for example, Bugnicourt et al., 2014; Liang et al., 2017; Seo et al., 2018) but appear quite efficient for bactericidal application (see for example, Ivanova et al., 2013 and Cheng Y. et al., 2019 for a review). We will present here some generic mammalian cell responses to representative examples of the wide repertoire of topographical cues explored in the literature, from classical unidirectional substrates (e.g., grooves) to multidirectional arrays. We will in addition review some more complex topographies, e.g., gradients, short-range asymmetrical cues, or fibrous substrates.

### Unidirectional Topographical Cues

Rectangular grooves of various dimensions constitute the most classical example of unidirectional cues, used by a great number of studies to investigate *in vitro* the phenomenon of contact guidance. These substrates are composed of parallel arrays of grooves separated by ridges, and can be referred to as grooves, ridges, or grating topography. These types of substrates aim at mimicking physiological topographies such as aligned fibers or ECM tracks that a variety of cells can encounter *in vivo* but more generally now serve as reference structures to assess cell responses to topographical cues. A number of studies have investigated the influence of grooves/ridges dimensions on cell morphology and orientation and have highlighted a strong influence of grooves width and depth on contact guidance. These founding papers in the field have been reviewed elsewhere (Kim et al., 2012; Nguyen et al., 2016). We will therefore recall here their most general results and conclusions, before reporting on recent studies implementing more elaborate unidirectional topographies (e.g., non rectangular grooves).

#### Cell Responses to Grooves

##### Morphological modifications

A majority of cells respond to parallel arrays of grooves and ridges by a clear alignment and elongation along the substrate main axis, in contrast to flat control surface where cells usually exhibit random orientation and more rounded morphologies (Table 1). Overall, the dimensions of topographical structures appear to be a key determinant of this cellular alignment. The most consistent trend corresponds to increased cell elongation and alignment for decreasing grooves/ridges width and increasing depth (Clark et al., 1990; den Braber et al., 1998; Walboomers et al., 2000; Teixeira et al., 2003; Charest et al., 2007; Loesberg et al., 2007; Fraser et al., 2008; Biela et al., 2009; Kim et al., 2009a; Franco et al., 2011). Above a critical size ranging from a few dozen to hundred micrometers, cells responses to grooves fade out and cell behaviors become similar to those observed on flat surfaces. Symmetrically, minimal size thresholds have been reported under which cell alignment is no longer observed (75 nm width and 33 nm depth for rat osteoblasts for instance, Lamers et al., 2010). Altogether, it seems to exist for each cell type an optimal range of topography dimensions that elicit the maximal response in terms of alignment and elongation. The strongest responses are usually seen when the cell can span over a few grooves/ridges, suggesting the existence of critical size and spacing of topographical sensors. In addition to the separate influence of the width and depth of grooves on cell alignment, the influence of the aspect ratio of grooves (ratio between groove depth and width) was also investigated. A monotonic increase of cell elongation and alignment with the aspect ratio was for instance noted for human mesenchymal stem cells (hMSCs) and human dermal fibroblasts (Crouch et al., 2008; Wong et al., 2014). Quite interestingly, perturbation of the keratin cytoskeleton in pancreatic cancer cells was shown to increase their morphological response to the grooves depth, while the response to the width and spacing was unchanged (Holle et al., 2017). These results show that the phenomena of cell elongation and alignment linked to contact guidance are not dependent on a single dimension and that cells are rather able to integrate a combination of cues along the three directions of space.

**TABLE 1:** Effect of grooved substrates on cell alignment and migration.

Cell type	Dimensions	Material	Cell shape and orientation	Cell migration	References
Corneal epithelial cells	Depth 600 nm Pitch 400 nm–4 μm	Silicon (SiOx)	// Elongation and alignment (∼35% of the cells). Increasing with groove depth		Teixeira et al., 2003
Keratocytes	Depth 600 nm Pitch 400 nm–4μm	Silicon (SiOx)	// Elongation and alignment (∼70% of cells). Decreased for smaller pitches		Teixeira et al., 2004
Human mesenchymal stem cells	Depth 300 nm Pitch 400–4000 nm	Polyurethane	// Elongation and alignment (∼70% of cells). Increased for 1400–4000 nm pitches		Watari et al., 2012
Myoblasts	Depth 5 μm Width and spacing 5–75 μm	Polycarbonate	// Alignment (∼60% of cells). Decreased with increasing width (optimal at 10 μm)		Charest et al., 2007
Oligodendrocytes	Depth 100–1200 nm Width 100–4000 nm Spacing 100–8000 nm	Quartz	// Alignment Decreasing with increased spacing		Webb et al., 1995
Rat hippocampal neurons	Depth 14–1100 nm Width 1–4 μm	Quartz	// Alignment on deep wide grooves. Perpendicular alignment to shallow narrow grooves		Rajnicek et al., 1997
Osteoblasts	Depth 50–150 nm Pitch 500 nm	Polystyrene	// Elongation and alignment (∼25% of cells for 150 nm depth). Maximal on deeper grooves	Migration in the direction of the grooves. Maximal on deeper grooves	Lenhert et al., 2005
Osteoblasts	Depth 1 μm Width 4 μm Spacing 6 μm	PDMS	// Elongation and alignment	Migration in the direction of the grooves. Increased cell speed on grooves	Tang et al., 2014
Endothelial cells (HUVEC)	Depth 300 nm Pitch 400–4000 nm	Polyurethane	// Alignment (∼60% of the cells). Maximal for pitches 800–1200 nm	Migration in the direction of the grooves. Speed increased compared to flat for high pitches	Liliensiek et al., 2010
Fibroblasts	Depth 400 nm Width 1–9 μm Spacing 1 μm	Polyurethane	// Elongation and alignment. Stronger on small pitches	Migration in the direction of the grooves. Maximal speed for intermediate pitches	Kim et al., 2009a
Fibroblasts	Depth 5–22 μm Width 6–30 μm Spacing 5–25 μm	Titanium	// Alignment but no clear effect on elongation	Migration in the direction of the grooves. No significant increase in speed	Kaiser et al., 2006
Periodontal ligament fibroblasts	Depth 100 nm Pitch 500 nm	Photoresist	// Elongation and alignment	Migration in the direction of the grooves. No significant increase in speed	Hamilton et al., 2010
Corneal epithelial cells	Pitch 400 nm–4 μm	Polyurethane	// Alignment (∼80% of cells)	Migration in the direction of the grooves. Maximum motility on the 1.6 μm pitch (slight increase compared to flat)	Diehl et al., 2005
Neutrophils	Depth 3–5 μm Width 6–14 μm Spacing 2 μm	Glass/polyimide	Elongation Stronger on narrow grooves (6 μm)	Migration in the direction of the grooves (95% of cells) 10 times higher speed compared to flat. Biphasic relationship of speed with ridge spacing	Tan and Saltzman, 2002

##### Cell migration

In addition to cell orientation and morphology, many studies also investigated the movement of cells on grooved substrates. When cultured on these substrates, many cell types (such as fibroblasts, epithelial cells, endothelial cells, osteoblasts, immune cells, or neurons) show a directional migration parallel to the grooves in contrast to essentially random movement observed on flat surfaces (Table 1; Webb et al., 1995; Dalton et al., 2001; Tan and Saltzman, 2002; Mello et al., 2003; Diehl et al., 2005; Lenhert et al., 2005; Gomez et al., 2007a; Tzvetkova-Chevolleau et al., 2008; Biela et al., 2009; Kim et al., 2009a; Liliensiek et al., 2010; Kwon et al., 2012). Many studies observed a globally increased cell motility on these microstructured substrates compared to flat surfaces (Tan and Saltzman, 2002; Mello et al., 2003). Cell motility also appears dependent on topography dimensions, with some studies reporting an influence of grooves/ridges spacing on cell speed (Tan and Saltzman, 2002; Kim et al., 2009a). In both studies, the highest cell motility was observed for the intermediate spacings in the range of dimensions chosen by the authors: 10 μm between ridges using neutrophils (Tan and Saltzman, 2002), between 5 and 6 μm for fibroblasts (Kim et al., 2009a).

#### Alternative Grooved Geometries

##### Sinusoidal grooves

One can argue against the physiological nature of the sharp edges present in most grooved substrates. An increasing number of studies therefore develop and investigate curved/wavy surfaces (Figure 1B). Long overlooked because of technical limitations, the influence of curvature on cell behavior is now the subject of active research (Bade et al., 2018; Liu et al., 2018; Pieuchot et al., 2018; Yip et al., 2018). We will mention here only a few of these studies, but for more details see the in-depth review of Baptista et al. (2019). On wavy surfaces with wavelengths (peak-to-peak distance) from 1.7 to 6.3 μm, myoblasts orient in the direction of the pattern, and show more pronounced alignment for increasing wavelengths (and therefore decreasing curvature) (Linke et al., 2019). Using larger wavelengths, Song et al. (2015) showed that T-cells preferentially localized to the concave regions of the pattern and displayed contact-guided migration in the direction of the grooves. Cell directionality and speed tended to decrease when the wavelength increased from 20 to 160 μm (Song et al., 2015). In a more recent study, Cheng D. et al. (2019) found similar results with vascular endothelial cells and interestingly observed that the cells still exhibited directional migration on grooves with wavelength as long as 150 μm, a length scale higher than the size of individual cells.

##### Multiscale grooved substrates

**FIGURE 2:**
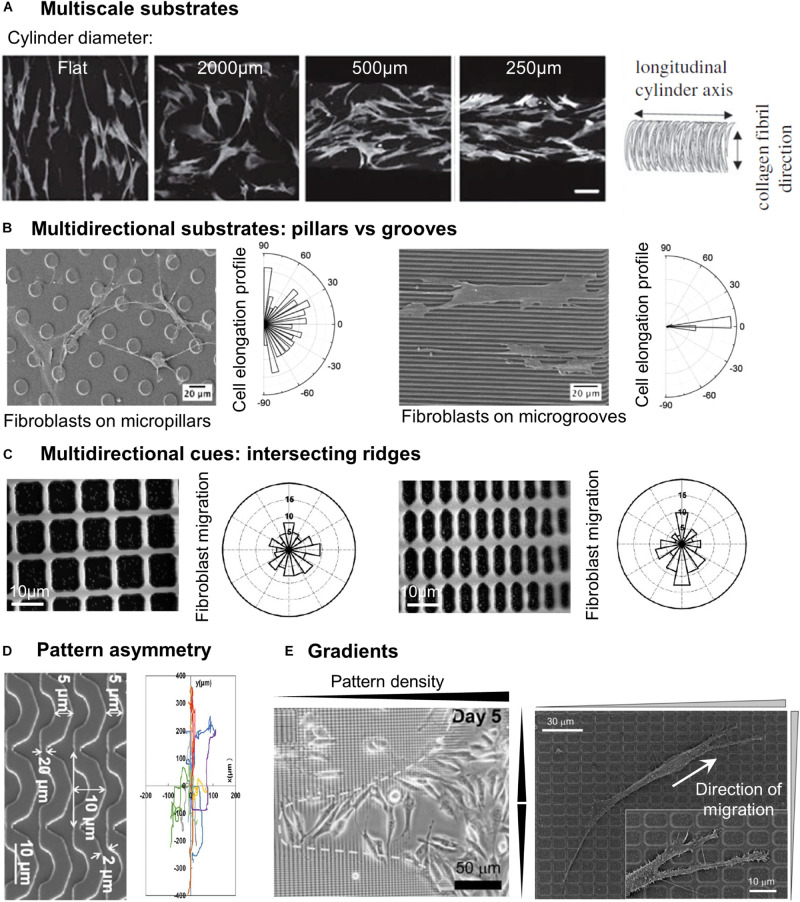
Cell response to different categories of microstructured substrates. **(A)** Human bone marrow stromal cells stained by F-actin on cylinders of different curvatures coated with anisotropically aligned collagen nanofibrils. Scale 100 μm. Reproduced with permission from Werner et al. (2018). **(B)** SEM images of fibroblasts on micropillars or microgrooves, associated with the cell elongation profile. Reprinted with permission from Ning et al. (2016). Copyright 2016 American Chemical Society. **(C)** Direction of fibroblast migration on a grid of intersecting ridges with different anisotropy. Reproduced with permission from Kim et al. (2009b). **(D)** Mouse osteoblastic cells migration tracks on substrates of arcs. Reproduced with permission from Tang et al. (2014). **(E)** Accumulation of fibroblasts in the center region of a gradient pattern with the highest density of structures (left). Close-up of a fibroblast migrating toward denser regions of the pattern (right). Reproduced with permission from Kim et al. (2009b).

*In vivo*, most cellular environments and adhesion substrates present a multiscale organization, with both nanometric and micrometric topographical cues. *In vitro*, the deposition of random nanometric topographical irregularities (colloidal particles) on micrometric grooves impaired the alignment of epithelial cells observed on smooth grooves (Andersson et al., 2003). This potential competition between nanometric and micrometric cues was more systematically investigated by combining micrometric grooves with arrays of nanometric grooves in different directions, i.e., parallel or perpendicular to the micrometric grooves. Nano and microgrooves in the same direction induced the highest level of alignment of rat mesenchymal stem cells (compared to nano or microgrooves alone), indicating a synergetic effect of the two topographical scales. On the contrary, the lowest alignment was observed when the two arrays were perpendicular, with cells responding to either one or the other scale of cues (López-Bosque et al., 2013). The same effect was observed on a system of aligned nanofibers on top of micrometric grooves with endothelial cells (Moffa et al., 2014). Other sets of studies combined nanometric topography with curved micrometric substrates, yielding slightly different results. In a first system, substrates with 250 nm wide grooves were compressed to generate micro- sinusoidal grooves (30/100 μm wavelength). When nano and micrometric cues were in the same direction, fibroblasts showed higher alignment and elongation than on the micrometric wrinkles alone. When nanogrooves were oriented at 45° of the wrinkles direction, cells orientation followed the nanometric array whereas random cell orientation was observed for a perpendicular orientation of the two topographical scales (Bae et al., 2015; Seonwoo et al., 2016). The authors of these two studies concluded overall that cells were more strongly influenced by nanometric cues. In a similar idea, Werner et al. (2018), developed a system of nanofibrils of collagen deposited perpendicularly to the long axis of cylinders of different curvatures. While on flat and low curvatures, human bone marrow mesenchymal stem cells (hBMSCs) orientation and migration was controlled by the nanometric collagen fibrils, the cells aligned and migrated along the cylinder axis for higher curvature (diameter <500 μm) (Figure 2A). The authors therefore concluded that in this situation, guidance by cues with dimension greater than the cell (here in the form of high curvature avoidance) can dominate over nanotopographical cues (Werner et al., 2018). The outcome of competing multiscale topographies is therefore likely dependent on the precise shape and dimension of the topography, as well as on the cell type.

### Multidirectional Topographical Cues

#### Discontinuous Multidirectional Cues: Pillars and Pits

A first class of multidirectional topographies used in the literature includes arrays of pillars of different shapes (i.e., round, square, or hexagonal: see, e.g., Micholt et al., 2013; Tang et al., 2014; Liang et al., 2017), dimensions and rotational or line symmetry (Figures 1E,F). Cell responses to multidirectional cues are usually quite different from those on unidirectional topographies (Table 2). In particular, in contrast to elongated morphologies observed on grooves, cells show branched morphologies when the characteristic dimensions of the pillared surface is in the micron range, matching the cell size (Frey et al., 2006; Ghibaudo et al., 2009; Ning et al., 2016; Figure 2B). Nanopillared surfaces also induce more random orientation and morphology compared to nanogrooves (Choi et al., 2007; Lamers et al., 2010; Ning et al., 2016). For both ranges of pillar dimensions, migrating cells show less directional movement compared to flat or grooved substrates (Frey et al., 2006; Lamers et al., 2010; Tang et al., 2014; Liang et al., 2017). The influence of multidirectional cues on cell motility is however less clear. Some studies have measured high cell motility on these substrates compared to nanogrooves (Lamers et al., 2010; Liang et al., 2017) or flat surfaces, while others noted the opposite result (Tan et al., 2000; Tang et al., 2014). Note that the aspect ratio of pillars is expected to change the effective substrate stiffness compared to the bulk material of which they are made (see for example, Fu et al., 2010 and Bugnicourt et al., 2014 for the mathematical relation between the Young modulus of the bulk material of a rod and its apparent Young modulus). The stability of arrays of such high-aspect ratio structures against adhesive and capillary forces becomes then an issue (see for instance the work of Albuschies and Vogel, 2013). For a careful analysis as well as examples of experimental achievements regarding stable high-aspect ratio pillared surfaces, in particular in the case of polymeric materials and hydrogels, the reader is referred to (Chandra and Yang, 2010). However, the relatively low aspect ratio of stable pillar arrays in the aforementioned studies (see Table 2), even for the softest material used (i.e., standard PDMS), excludes any contribution of a stiffness-associated mechanosensing.

**TABLE 2:** Cell responses to anisotropic versus isotropic topographies.

Cell type	Type of topography	Material	Dimensions	Cell response	References
Fibroblasts	Grooves	Silicon	Short (50–100 nm height) Medium (200–300 nm) Tall (500–600 nm) Pitch 230 nm	Clear alignment and elongation // to grooves. Increasing with increasing height	Choi et al., 2007
	Posts			Some elongation. Decreasing with increasing height	
Osteoblasts	Grooves	PDMS	Depth 1 μm Width 4 μm Spacing 6 μm	Migration // to grooves	Tang et al., 2014
	Pillars		Height 1 μm Size 10 μm	Random migration. Lowest speed	
Fibroblasts	Grooves	PDMS	Depth 800 nm Width and spacing 750 nm	Migration // to grooves	Tzvetkova-Chevolleau et al., 2008
	Pillars		Height 800 nm Diameter 1 μm Spacing 1.6 μm	More random migration, although some preferential movement at 0 and 90°	
Osteoblasts	Grooves	Polystyrene	Depth 10–150 nm Pitch 80–1000 nm	// Elongation and alignment, increasing with increasing pitch and depth Random migration	Lamers et al., 2010
	Pillars		Height ∼120 nm Diameter ∼300 nm Spacing 80–1000 nm	Random orientation. Random migration, high motility	
Fibroblasts	Grooves	Polymethyl methacrylate (PMMA)	Depth 300 nm Pitch 860 nm	Migration // to the grooves Low motility	Liang et al., 2017
	Pillars		Height 300 nm Width 70–430 nm Spacing 200–700 nm	More random migration. High motility, maximal for small spacings	
Glioma cells and fibroblasts	Grooves	Polyacrylonitrile (PAN)	Height 1.2 μm Width 2.4 μm Spacing 2 μm	// Alignment and elongation for both cell lines (but glioma cells less elongated)	Ning et al., 2016
	Pillars		Height 1.5 μm Width 12 μm Spacing 25 μm	No elongation or orientation	

Arrays of holes (Figure 1D) generate different and sometimes opposite effects. Tan et al. (2000) observe both decreased adhesion and increased neutrophils motility on a 10 μm square array of 2 μm diameter micrometric holes compared to flat or mirror pillared surfaces (Tan et al., 2000). Using deeper holes (5 μm), Berry et al. (2004) show that the effect of pits on fibroblast migration is dependent on the pattern depth, i.e., on the cell ability to achieve sufficient deformation to adhere on the bottom surface of the pits. The highest motility is measured on the smallest pits (7 μm diameter, 20 μm spacing) where cells can span above holes rather than conforming to the vertical edges of larger holes (Berry et al., 2004). The limitation of the surface available to cell adhesion appears also critical using nanometric holes. By generating pores of 40 or 80 nm in diameter inside aluminum oxide membranes (pitch of about 100 nm in both cases), Nasrollahi et al. (2017) observed faster and more persistent fibroblasts migration on the larger pores, i.e., on the smallest available surface for cell adhesion. In another study, Jeon et al. (2015) used substrates with zones of different densities of nanometric pits, and observed that fibroblasts migrated away from regions of high pits density. The authors interpreted this result as a cell-repellant effect of holes, but it may also be the result of an increased motility of cells on the area of high pit density, in agreement with the observations of Nasrollahi et al. (2017).

#### Continuous Multidirectional Cues

While most of the multidirectional patterns are provided to cells as discontinuous cues, cells can also experience multidirectional cues in the form of continuous structures, for instance intersecting ridges (Figure 1C). Following this idea, Mai et al. (2007) tested the response of smooth muscle cells to multidirectional cues by designing a series of grid patterns: perpendicular arrays of ridges enclosing square (fourfold symmetry) or rectangular (twofold symmetry) holes in the micron range of increasing aspect ratio, providing different substrates characterized by distinct densities of guiding ridges in the two orthogonal directions. The cellular responses to parallel ridges were reported for comparison (Mai et al., 2007). Similar to the reverse protrusive structures (e.g., square pillars), smooth muscle cells orient randomly on the squared array of ridges. Interestingly, cells cultured on rectangular grids align in the direction of the longer side of the grid, in other terms they elongate along the ridges presenting the longest distance in-between the intersections. Similar results were obtained with fibroblasts, which elongated and migrated along the long axis of a grid pattern (Kim et al., 2009b; Figure 2C). However, the introduction of gaps (i.e., discontinuities) in the corners of such a grid of ridges were shown to guide cell elongation diagonally through the gaps (Hamilton and Brunette, 2005), illustrating the sensitivity of cells to the continuous or discontinuous nature of the topography.

More generally, these results reveal that cells are able to integrate simultaneously different multidirectional cues and selectively respond to the dominant one.

### Complex Topographical Cues

In addition to the classical patterns of grooves or pillars, a number of studies have challenged cells with more elaborate or more physiological topographical designs. In doing so, they have demonstrated the exquisite sensitivity of cells to contact guidance and their ability to integrate complex spatial information.

#### Local Asymmetry

Tang et al. (2014) tested the influence of the pattern symmetry by using three types of topographies with different rotational symmetries: arrays of square pillars and parallel grooves exhibiting fourfold and twofold rotational symmetry, respectively, as well as a pattern of “arcs” which consists of connected repeating units providing local asymmetry (Figure 2D). Interestingly, mouse osteoblastic cells migrating on this latter most asymmetrical topography showed the highest motility as well as the strongest directional persistence (Tang et al., 2014). It was hypothesized that this microenvironment asymmetry was responsible for a shape asymmetry between cell front and back, sustaining cell polarization and directionality. In the same spirit, Sun et al. (2015) showed using arrays of sawtooth ridges that repeated local asymmetries at the subcellular scale were able to generate a unidirectional movement dependent on the characteristic size of the sawtooth.

#### Topographical Gradients – Topotaxis

Some studies also showed that cells are able to detect and respond to gradients of both unidirectional or multidirectional topographical cues. By using a lattice network of orthogonal ridges with increasing densities (i.e., decreasing spacing) toward the center of the pattern (Figure 1), Kim et al. (2009b) showed that fibroblasts migrated toward the higher densities regions, therefore accumulating in the center of the pattern (Figure 2E). In another study, the same group used patterns of parallel ridges with a gradient of spacing (Figure 1), and showed that fibroblasts tend to migrate toward the center of the pattern with intermediate ridge spacing of around 6 μm (Kim et al., 2009a). Similarly, Park J. S. et al. (2016) observed the migration of melanoma cells in an array of posts with a gradient of spacing in one direction (Figure 1) and showed directed migration toward sparser regions for invasive melanoma cells, but a reverse migration for non-invasive melanoma cells. They named this migration guided by density of topographic features “topotaxis” (Park et al., 2018). *In vivo*, topotaxis could account for different migration of cancer cells or certain aspects of wound-healing processes in response to different densities of ECM elements.

#### Electrospun Fibrous Substrates

Synthetic fibrous substrates aim at mimicking the fibrous nature of the ECM. Electrospinning is a widely used method that creates micro to nanofibers by ejecting a polymer melt or solution through a spinneret under a high-voltage electric field and that solidify or coagulate it to form a filament. The collected fibers can be left with a random orientation or aligned by different methods (Yuan et al., 2017) thereby creating a complex (but more physiological) topographical environment that presents either unidirectional or multidirectional cues to cells. More recently, electrospun shape memory polymer scaffolds that can change fiber alignment on command have been developed, alternatively presenting both types of cues to the same cells (Wang et al., 2017).

In line with the behavior observed on unidirectional grooved substrates, cells including mesenchymal stem cells (MSC) (Bashur et al., 2009; Subramony et al., 2013), endothelial cells (Whited and Rylander, 2014; Li et al., 2018), glioblastoma cells (Kievit et al., 2013), or astrocytoma cells (Vimal et al., 2016) adopt on aligned fibers an elongated, spindle-like morphology and align along the fiber direction. Accordingly, cells migrate in the direction of the fibers (Kievit et al., 2013; Lee et al., 2014), faster and longer distances compared to randomly oriented fibers (Lee et al., 2014; Mi et al., 2015; Azatov et al., 2017; Wang et al., 2017). Fibers alignment can also have a cell-type dependent effect on cell proliferation: while fibroblasts (Lee et al., 2017) or corneal epithelial cells show an increased proliferation on non-aligned fibers (Yan et al., 2012), keratocytes (Yan et al., 2012) and MSC proliferate more on aligned scaffolds (Subramony et al., 2013). Alignment of fibers has also been shown to improve myogenic (Abarzúa-Illanes et al., 2017) or neural differentiation (Wang et al., 2012; Abbasi et al., 2016; Lins et al., 2017) for instance compared to randomly organized fibers environments.

Fibers diameter has also been proved to influence cell morphology and migration. The elongation, alignment, and area of human MSC or fibroblasts is more pronounced on larger, microfibers (1–2 μm) compared to smaller nanofibers (80–740 nm) (Bashur et al., 2009; Chaurey et al., 2012; Lee et al., 2017). Accordingly, the trajectories of single neuronal ND7/23 cells tightly followed micrometric (5 μm) fibers orientation but were independent of submicrometric (0.74 μm) fibers orientation (Binder et al., 2013). One explanation for these observations is the fact that on small, nanometric fibers a single cell is more likely to interact with multiple fibers, thereby creating more variation in cell orientation and movement. Nevertheless, a couple of studies have reported an increased migration velocity on nanofibers (200–700 nm) compared to microfibers (1.1–5.7 μm) (Binder et al., 2013; Kievit et al., 2013; Meehan and Nain, 2014).

Another parameter to take into account is the size of the pores created by the space in between the fibers, which is modulated by the fiber’s density. While human MSC align more on the highest density of aligned fibers (interfibrillar distance around 10 μm) (Chang et al., 2013), endothelial cells migrate longer distances on low density scaffolds (Bouta et al., 2011). Indeed, when pore size in between fibers increases cells can only migrate on a single fiber (Lowery et al., 2010), leading to increased cell velocities.

Because of their physiological properties and their capacity to control various cell functions, biomimetic fibrous scaffolds now constitute very promising strategies for tissue bioengineering applications (Wang et al., 2013). In addition, recent technological advances in electrospinning now enables the design of complex and multiscale architectures (Robinson et al., 2019).

### In Brief: General Rules Governing Contact Guidance and Beyond

The development of microfabrication techniques has enabled the design of a wide variety of artificial topographies. We have tried in this section to provide a general classification of these topographies based on their degree of symmetry combined with the continuous or discontinuous nature of the cues, as illustrated in Figure 1. We would like to point out that some of these classifications can be subjective. For instance, what is classified as intersecting ridges by some authors might also be seen as arrays of square or rectangular holes. Conversely, close pores might be considered as a pattern of ridges. This subjectivity reflects the need to identify, for each case, the structures on which cell develop adhesive contacts underlying the contact guidance phenomenon.

Overall, most studies tend to report cell elongation, alignment, and guidance along unidirectional continuous cues, in contrast to more complex (i.e., branching) and less directed responses (i.e., random migration) on multidirectional discontinuous cues. In addition, many reports in the literature have highlighted cells’ ability to provide an integrated response when subjected to cues of different orientations or sizes along the three directions of space. However, most geometrical parameters of substrate topography (shape, size, area, orientation, curvature, aspect ratio, etc.) being linked, it is often impossible to clearly associate a particular cell response to a single topographical feature.

More globally, the strong dependence of contact guidance on substrates dimensions might suggest some kind of matching between cell characteristic sizes and the dimensions of the topographical cues, governing cellular responses across different topographies. Given the great variety of cell shapes and associated subcellular structures, this matching effect is likely to be cell-type dependent. More generally, the discrepancy between the modalities of contact guidance sometimes reported in the literature for similar topographical cues might arise from the specificities of the cells, e.g., their shapes or modes of migration. This will be reviewed in the following section. We can also point out the difficulty in most studies to deconvolute the pure effect of topography from other factors such as substrate stiffness, surface chemistry, protein coating, etc. The complex combination of all these factors is likely to drive the observed cell response and can also explain the variability of such responses in the literature.

## Cell-Type Dependent Response to Topography

Cells from multicellular organisms exhibit a large variety of functions related to their parent tissue, morphology or mode of migration. Whether it exists a cell-type dependent response to topography therefore appears like a central question. We will restrict our interest to the most studied topographies, i.e., grooves or pillars, in order to highlight the specificities of the cellular responses they trigger. The mode of migration recapitulates a few important traits of a cell, including adhesion and contractility. Some of the literature focusing on the consequences of mesenchymal versus ameboid cell properties on contact guidance will be reviewed in a first section. We will then dedicate a second section to neurons, whose micron sized extended processes and branched morphology has inspired numerous studies revealing interesting and new facets of the phenomenon of contact guidance.

### Non-neuronal Cells Response

Numerous studies have demonstrated cell type specific responses to topography, which can partly explain some of the discrepancies observed in cell response to similar topographies, as briefly mentioned in the previous section. By simultaneously observing the behavior of different cell types in similar microstructured environment, several studies report differences in the extent of alignment and contact guided migration, or in the range of topography dimensions eliciting cell responses (Table 3). Different elements can be put forward to explain these cell type specific responses such as the intrinsic morphology and/or variations in the intracellular organization associated with their physiological function.

**TABLE 3:** Cell type specific responses to grooved substrates.

Cell type	Dimensions	Cell shape and orientation	Cell migration	References
Human umbilical vein endothelial cells (HUVEC)	Depth 300 nm Pitch 400–4000 nm	// Alignment (∼60% of the cells). Maximal for pitches 800–1200 nm	Highest contact guided migration in the direction of the grooves. Highest motility	Liliensiek et al., 2010
Human dermal microvascular endothelial cells (HmVEC-d)		// Alignment for every pattern (∼50%). Maximal for 4 μm pitch (70%)	Intermediate contact guided migration. Low motility	
Human aortic endothelial cells (HAEC)		// Alignment (∼60% of the cells). Decreased for 400 nm pitch	Lowest contact guided migration. Low motility	
Human saphenous vein endothelial cells (HSaVEC-c)		// Alignment (∼60% of the cells). Decreased for 400 nm pitch	Intermediate contact guided migration. Intermediate motility	
Fibroblasts	Depth 200–500 nm Width 2–10 μm	Strongest elongation and alignment // to the grooves. Increased with decreasing width and increasing depth	Migration in the direction of the grooves, strongest	Biela et al., 2009
Endothelial cells		// Alignment, lowest elongation. Increased with decreasing width	Migration in the direction of the grooves, milder	
Smooth muscle cells		// Alignment, intermediate elongation. Increased with decreasing width	Migration in the direction of the grooves, milder	
Corneal keratocytes	Depth 300 nm Pitch 400 nm–4 μm	// Elongation and alignment (∼60% of cells). Increased with pitch (>800 nm)	Very little migration	Pot et al., 2010
Fibroblasts		// Elongation and alignment (∼70% of cells). Increased with pitch (>800 nm)	Migration // to grooves Slight motility increase compared to flat. Biphasic relationship between pitch and motility (max at 1200 nm pitch)	
Myofibroblasts		// Elongation and alignment (∼50% of cells). Increased with pitch (>800 nm)	Migration // to grooves Slight motility increase compared to flat	
Fibroblasts (mesenchymal)	Depth 800 nm Width and spacing 750 nm	// Elongation and alignment	Migration // to grooves	Tzvetkova-Chevolleau et al., 2008
Fibrosarcoma cells (more amoeboid)		Less elongation	Weaker response	
T Lymphocytes (PBTL)	Depth 3.5 μm Width 10 μm	// Alignment	Migration // to grooves	Mello et al., 2003
T Lymphomas (HUT78)		Less alignment	Weaker directionality along grooves but similar speed	
MDA-MB-231 (breast carcinoma, mesenchymal)	Depth 600 nm Width and spacing 800 nm	// Alignment and elongation	Migration // to grooves. Strong contact guidance and high motility	Ray et al., 2017
T47D (breast carcinoma, epithelial)		Less alignment	Lower contact guidance and motility	
Corneal epithelial cells	Depth 70–800 nm Pitch 400–4000 nm	// Elongation and alignment (max 50% of cells). Increased with depth but not pitch		Fraser et al., 2008
Stromal fibroblasts		// Elongation and alignment (max 90% of cells). Increased with depth and at intermediate pitches 1200–1600 nm		

Some studies have investigated for instance the response to topography of different cell types found in the vascular system. Liliensiek et al. (2010) observed distinct orientation and elongation of vascular endothelial cells from various anatomical sites when cultured on micrometric and sub-micrometric grooves and holes. These differences are possibly attributable to intrinsic endothelial heterogeneity between tissues as well as different biophysical cues at the different sites (Liliensiek et al., 2010). Similarly, Biela et al. (2009) investigated the response of three cell types present within blood vessels (and possibly in contact with implant surfaces in the context of cardiovascular diseases): fibroblasts, endothelial cells, and smooth muscle cells, and noted a stronger alignment, elongation and directional migration of fibroblasts along the grooves. They hypothesized that these differences might be explained by the distinct functions of these cells types within the vascular system (Biela et al., 2009).

It has also been suggested that the response of single cells to the topography can depend on their mode of migration. The migration of isolated cells is usually schematically separated into two main modes: mesenchymal migration (characterized by a polarized cell morphology and movement at intermediate/low speed) and amoeboid migration (characterized by a round morphology, and a fast movement involving a strong cell contractility and low adhesion to the substrate). More generally, in terms of morphological changes, alignment, elongation, or directional migration, it appears that cells with a mesenchymal mode of migration exhibit a more robust response to topographical cues than cells that undergo amoeboid migration (highlighting the importance of integrin-based adhesion in contact guidance, see section “Topography Sensing at the Subcellular Level”). Accordingly, a strong response of fibroblasts to substrate topography has been reported across various studies, compared to other cell types (Fraser et al., 2008; Biela et al., 2009; Pot et al., 2010). Similarly, Tzvetkova-Chevolleau et al. (2008) observed a robust contact guidance of 3T3 fibroblasts along nanogrooves, but a milder response of fibrocarcinoma cells. The authors hypothesized that this difference could be due in part to the different modes of migration of these cells, with fibrocarcinoma cells exhibiting more rounded morphologies and amoeboid migration (Tzvetkova-Chevolleau et al., 2008).

Later, similar results were found with two different breast cancer cell lines: MDA-MB-231 cells characterized by a mesenchymal mode of migration and MTLn3 cells by an amoeboid mode of migration. When cultured on aligned type I collagen fibrils, MDA-MB-231 cells show a strong contact guidance, elongating, and migrating along the fibrils. In contrast, MTLn3 do not interpret the aligned fibrils as a contact guidance cue and exhibit random migration (Wang et al., 2014). More recently, this result was elegantly confirmed by showing an attenuated response of cells with an amoeboid phenotype to nanogrooves compared to another fraction of cells from the same pancreas adenocarcinoma line with a mesenchymal phenotype (Ray et al., 2017). One of the mechanisms explaining the different responses between mesenchymal and amoeboid migration can lie in the different degrees of cell adhesion to the substrate. This implies that mesenchymal cells would show maximal contact guidance response for cues dimension close to FA size, while amoeboid cells could respond more strongly to whole cell confinement.

### Response of Neuronal Cells to the Topography

Amongst different cell types in the body, neurons possess a unique branched and polarized morphology. Typically, the dimension of neuron cell bodies is on the scale of tens of microns, while thin and long branched neuronal processes (or neurites) in humans have diameters ranging from 0.01 to a few microns and lengths up to several tens of centimeters. Because of this variety of subcellular compartments in terms of size and shape, neurons exhibit specific responses to topography. One of the first demonstration of contact guidance was established on nerve fiber extensions guided by the oriented ultrastructure of a blood clot (Weiss, 1934). Since then, the influence of topographical cues on a variety of neuronal processes has been demonstrated, from early polarization events to neurite growth or branching.

#### Guidance of Neurite Growth by Grooves

**FIGURE 3:**
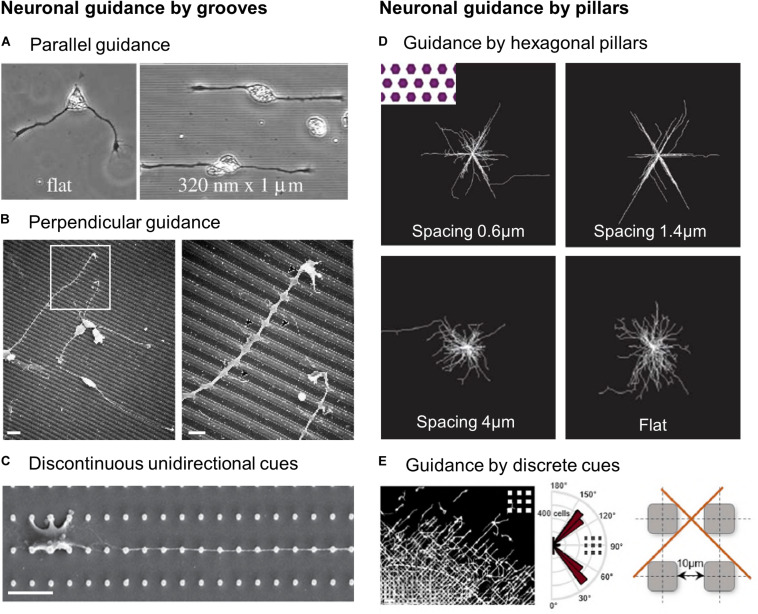
Guidance of neuronal cells by microstructured substrates. **(A)** Xenopus spinal cord neurons on flat or 1 μm wide, 320 nm deep grooves. Reproduced with permission from Rajnicek et al. (1997). **(B)** Scanning electron microscopy (SEM) images of cortical neuroblasts on grooves (1 μm width, 0.5 μm depth). Scales 5 μm (left), 2 μm (right). Reproduced with permission from Nagata et al. (1993). **(C)** SEM image of a primary hippocampal neurons on unidirectional micropillars array. Scale 10 μm. Reproduced with permission from Park M. et al. (2016). **(D)** Hippocampal neurons alignment on flat or in between hexagonal pillars of various spacing. Reproduced with permission from Kundu et al. (2013). **(E)** Alignment of migrating cortical interneurons in between square pillars. Scale 50 μm. Reproduced with permission from Leclech et al. (2019).

Similar to other cell types, substrates of grooves can guide neurons and in particular their neurites (Figure 3A). This effect is here also dependent on the dimension of the topography, i.e., groove depth and width. Nevertheless, probably due to their particular shape and small diameter, neurites have been shown to respond to very small grooves, as shallow as 14 nm (Rajnicek et al., 1997). Most studies describe an increased neurite response in terms of outgrowth and alignment with increasing groove depth (ranging from 0.2 to 4 μm) (Hirono et al., 1988; Clark et al., 1990; Rajnicek et al., 1997; Miller et al., 2002; Gomez et al., 2007b; Chua et al., 2014). This effect was also observed on irregular edges of grooves generated by laser scribing onto a graphene oxide surface (Lee et al., 2018). The influence of the width of groove or ridge appears more complex, with contradictory results according to the dimensions of the topography and types of cells used (Yao et al., 2008; Ferrari et al., 2011; Chua et al., 2014).

#### Contact Guidance Specificities of Neuronal Cells

##### Perpendicular contact guidance

While most neurons, similar to non-neuronal cells, grow, and align in the direction of the topography main axis (a phenomenon also named “parallel contact guidance,” Figure 3A), an alternative behavior designated as “perpendicular guidance,” where neurites elongate perpendicularly to grooves, is also regularly reported (Figure 3B). Once again, this process appears dependent on groove dimensions: perpendicular guidance of hippocampal neurons was predominantly observed on the narrowest (i.e., 1 μm) grooves (as shallow as 14 nm) while parallel guidance was achieved for wider and deeper grooves (4 μm wide, 1 μm deep) (Rajnicek et al., 1997). In agreement with this observation, decreasing groove width (from 2 μm to 300 nm) at a constant depth (450 nm) promoted more perpendicular alignment in the same type of neurons (Fozdar et al., 2010). In the same line, Chua et al. (2014) reported a decreasing fraction and length of perpendicularly oriented neurites of fully differentiated murine neural progenitors with increasing groove depth (from 0.35 to 4 μm). An interesting effect of depth was noted for large grooves (50–350 μm): axons turned at the edge of deep grooves (22–69 μm), but crossed shallow ones (2.5–11 μm). From a dynamic point of view, this phenomenon also appeared to depend on the angle at which the neurite approaches the edge: above a threshold height of 11 μm, cortical neurites approaching perpendicularly tend to keep their direction while neurites arriving with a more parallel orientation often turn to follow the groove (Li and Folch, 2005). The phenomenon of perpendicular guidance also depends on neuron types and species as it has been observed for primary hippocampal neurons in rats (Rajnicek et al., 1997; Gomez et al., 2007b; Fozdar et al., 2010) but not xenopus (Rajnicek et al., 1997) and in central nervous system neuroblasts but not in peripheral ones (Nagata et al., 1993). Interestingly, the incidence of perpendicular guidance was also reported to be dependent on the age of the embryo from which neurons were harvested, suggesting a developmental regulation of this process (Rajnicek et al., 1997). Perpendicular guidance was also reported on dorsal root ganglia (DRG) explants cultured on aligned nanofibers *in vitro* (Xie et al., 2014). Similarly, an electrospun nanofibrous scaffold implanted into a rat brain elicited perpendicular neurite growth rather than parallel (Nisbet et al., 2009). Interestingly, the process of perpendicular contact guidance has not only been described *in vitro*, but also *in vivo* for instance in the cerebellum where vertically migrating granule cells interact with tangential parallel fibers (Ono et al., 1997). It is also worth mentioning that few cases of perpendicular guidance have also been reported for non-neuronal cells (Xu et al., 2018).

##### Neurite guidance by multidirectional cues

Unlike most other cell types, neurons and their processes exhibit particular and fascinating responses to multidirectional cues. These topographies have been presented to neurons mostly in the form of regular lattices of micron-sized pillars that have different shapes and dimensions. These types of substrates can be separated according to their dimensions: structures and spacing at the micrometer scale, where neurites grow in between the structures, or dense arrays of nanometric structures where neurites are navigating on top of the structures. Both can support the elongation of hippocampal neuronal processes, resulting in longer neurites compared to flat surfaces (Dowell-Mesfin et al., 2004; Micholt et al., 2013; Park M. et al., 2016). With micrometric structures, pillar size, and most importantly spacing influence neurite outgrowth and alignment (Dowell-Mesfin et al., 2004; Hanson et al., 2009; Kundu et al., 2013): big posts with features size from 10 to 100 μm were shown to influence neurite outgrowth although a stronger alignment was observed with the smallest structures and spacing (10 × 10 μm) (Hanson et al., 2009). With smaller pillars (2 μm diameter, 1 μm height), hippocampal neurites show the highest alignment for the smallest spacing of 1.5 μm. As the spacing increased, the neurites orientations were getting closer to the random pattern present on flat surfaces (Dowell-Mesfin et al., 2004). A comparable result was found by Kundu et al. (2013) who observed that hippocampal neurites among hexagonal pillars grew along three preferential orientations, and that the highest guidance precision was obtained for an optimal spacing of 1.4 μm (Figure 3D). We can point out that this size is close to the axon dimension, arguing once again in favor of a matching between cell structures and topographical cues dimensions. A higher degree of guidance and oriented growth can be achieved by introducing some anisotropy in the pattern. Indeed, starting from a homogeneously spaced, fourfold symmetrical array (i.e., with four axes of symmetry), increasing the spacing of pillars in one direction creates a more unidirectional pattern characterized by parallel rows of pillars (Figure 3C). For various size of structures this resulted in an important directional neurite growth oriented along the rows of pillars, even more pronounced when increasing the row spacing, i.e., when preventing neurites from reaching the neighboring rows (Prinz et al., 2008; Kundu et al., 2013; Park M. et al., 2016; Figure 3C). Along the same lines, Simitzi et al. (2015) observed that arrays of aligned elliptical microcones (therefore with an intrinsic anisotropy) elicited a more oriented growth of sympathetic axons compared to a randomly organized lattice of round cones.

#### Unifying View: Neuronal Guidance by Successive Discrete Cues

It is interesting to notice that while continuous unidirectional topographies such as grooves tend to decrease the complexity of the branching pattern of neurons (Hirono et al., 1988; Chua et al., 2014), multidirectional or isotropic patterns have the opposite effect. Indeed, neurites can occasionally branch at pillar intersections and secondary neurites can themselves be guided by the structures (Dowell-Mesfin et al., 2004; Hanson et al., 2009). More recently, it was clearly demonstrated that arrays of nanopillars increased the number of axon collateral branches in hippocampal neurons (Seo et al., 2018). Overall it seems that neurites tend to follow successive and discrete structures, and if another contact is nearby, they extend to reach the new location, resulting in aligned and branched neuritic arbors. It would be interesting to define more precisely the range of distances between contact points leading to such guidance, and whether this depends on intrinsic cellular determinants (cell dimension, directional persistence in neurite elongation, etc.).

Interestingly, this discrete and periodic guidance provided by discontinuous topographies is reminiscent of the phenomenon of perpendicular contact guidance observed on grooves. When crossing perpendicularly grooves, or when growing on top of pillars, neurites encounter a succession of discrete points of contact. Alternatively, neurites navigating on the bottom surface in between pillars find additional side adhesion by making periodic contacts with the edges of topographical cues. This type of guidance seems to be particularly potent and specific for the growth of long and thin structures such as neurites. This idea was indirectly confirmed by a study of Bucaro et al. (2012) who showed that several types of stem cells specifically develop neuronal morphologies with long axon-like processes on nanopillar arrays. More recently, a similar mechanism of contact guidance by discrete cues was observed in neuronal migration. Embryonic interneurons migrating in between 10 μm square pillars exhibit a striking alignment and directional migration along the diagonal direction of the square pillars array (Figure 3E). The interaction with the successive corners of the pillars was able to guide the elongation of straight neuronal leading processes. This behavior was abolished in an array of round pillars allowing extended adhesive contacts of the leading process around their circumference, almost suppressing the discrete contact guidance effect provided by square pillars (Leclech et al., 2019).

Lastly, we note that neurite guidance by topographical cues is most of the time associated with enhanced growth and, when studied, to accelerated polarization (Gomez et al., 2007b; Kang et al., 2012, 2016; Bugnicourt et al., 2014). Although no mechanism was clearly established, Micholt et al. (2013) have suggested that contacts with an array of pillars may provide multiple enhanced adhesion sites from which neurites could exert mechanical pulling forces. Considering that pulling on a neurite may trigger accelerated growth, this hypothesis would lead to a mechanically based interpretation of neurite response to topographical cues. Such a view is however not incompatible with molecular signaling induced at reinforced adhesion sites, providing regular boosts of neurite growth.

To conclude this entire section, the cell type specific response to topography is a multifactorial question probably involving a complex interplay between cell intrinsic characteristics associated with specific topographical cues in their native environment. The observations associated with this question can be exploited toward various applications, for example, the design of cell-type specific implant surfaces.

## Topography Sensing at the Subcellular Level

A large body of work describing cellular responses to nano or micrometric topographical cues is today available, as reviewed in the previous sections. The size of these cues is usually much smaller than the dimensions of the cell, which suggests than cells should possess structures at the submicron scale acting as sensors of their physical microenvironment. Cells or stable cell protrusions (i.e., microtubule-based, like neurites) are indeed able to generate dynamic and actin-based subcellular processes at different locations, in particular at their front where the tasks of exploring the microenvironment are performed (Ridley, 2011). In addition, contact guidance relies by essence on adhesive structures, at least in non-ameboid cells. Adhesion molecules can organize to form clusters named focal adhesions (FAs) whose size can match those of topographical cues, giving them a putative role as topographic sensors.

We will here review the literature focusing on the interactions of various subcellular structures with topographical cues. Once again, we will dedicate an entire sub-section to neurons. Indeed, neurites explore their environment through a specialized and highly regulated structure localized at their tip, the growth cone, which is crucial for apprehending neuron response to topography and that offers an interesting perspective on the phenomenon of contact guidance in general.

### Filopodia and Protrusions

Filopodia are thin cytoplasmic dynamic projections containing parallel cross-linked bundles of actin. They usually emerge from more extended but thin actin-rich and dynamic structures named lamellipodia. While filopodia were first described in living cells in 1961 (Gustafson and Wolpert, 1961), their substrate-exploring functions were suggested in 1976 and assumed to be regulated by mechanical forces (Albrecht-Buehler, 1976). They have since then been proved to be major sensing organelles of the extracellular environment, in particular for migrating cells, and as such can play a crucial role in the sensing and subsequent response to the topography.

#### Filopodia as the Primary Sensors of Topography

**FIGURE 4:**
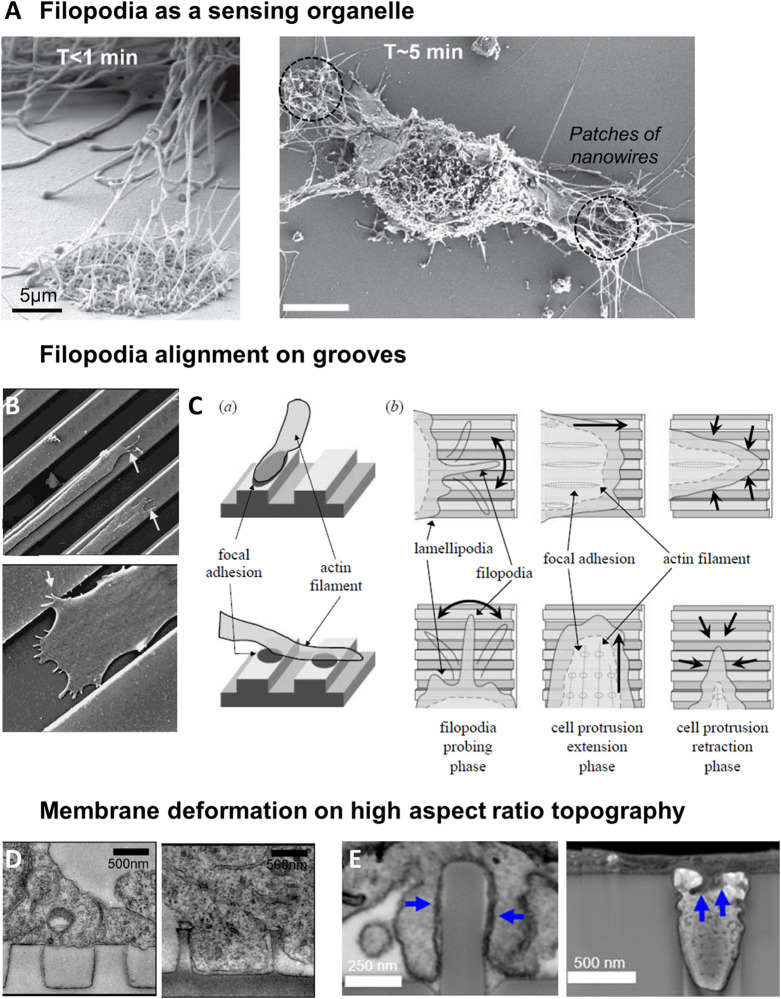
Filopodia as a sensor of topography. **(A)** Sensing of nanowires by filopodia of fibroblasts. Shortly after plating, filopodia explore both flat and nantopography (left). After 5 min, filopodia on flat surface have retracted (right). From Albuschies and Vogel (2013). **(B)** SEM image of epithelial cell sending filopodia along ridges and grooves. Reproduced with permission from Dalton et al. (2001). **(C)** Model of cell alignment on grooves by filopodia probing and perpendicular filopodia retraction. Reproduced with permission from Fujita et al. (2009). **(D)** Membrane deformation of cortical neurons on nanopillars seen by TEM. On pillars of 500 nm diameter, 1 μm spacing, cell body rests on top of the pillars (left). On 200 nm diameter pillars with the same center-to-center distance, membrane deforms to engulf the pillars (right). Reproduced with permission from Hanson et al. (2012). Copyright 2012 American Chemical Society. **(E)** FIB SEM cross sections reveal that plasma membrane wraps tightly around nanopillars with 400 nm diameter (left), but hardly deforms into 400 nm diameter nanopores (right, blue arrows). Reproduced with permission from Santoro et al. (2017). Copyright 2017 American Chemical Society.

Many studies have reported an active and fast probing of the extracellular environment by filopodia, which act as a scaffold to guide the growth of lamellipodial extensions (Fujita et al., 2009; You et al., 2014). While quickly retracted on flat surfaces, filopodia of fibroblasts have been seen to establish long lasting interactions with patches of silicon nanowires within the first minutes after cell seeding (Figure 4A; Albuschies and Vogel, 2013). More generally, it was thus observed that cells tend to present more filipodia on nanostructured compared to flat surfaces (Dalby et al., 2004a,b,c; Brammer et al., 2008). Filopodia can probe topographical cues of very small size: they have been shown to sense pits down to a diameter of 35 nm and a depth of 50 nm (Dalby et al., 2004a), or grooves as shallow as 71 nm (Wójciak-Stothard et al., 1996). Nevertheless, their interaction with topography appears to decrease with decreasing size of topographical cues from approximately a hundred to a dozen nanometers (Dalby et al., 2002, 2004a). This result can be interpreted as a scaling of the dimension of the spatial cues detected with the size of filopodia (100–300 nm). Supporting this hypothesis, Sales et al. (2017) have measured randomly oriented and shorter filopodia on micrometric grooves compared to flat surfaces and suggested that these structures were not essential for the response to microscale features. In these environments, lamellipodial structures may be instead the driving element of contact guidance (Sales et al., 2017). It is also interesting to mention that in hippocampal neurons cultured on nanobeads, the lower limit of bead size which elicit neurite response corresponds to the size of filopodia, while the upper limit is around the diameter of the axon (Kang et al., 2014).

#### Role of Filopodia and Cell Protrusions in the Response to Grooves

Restriction of lateral protrusions in migrating cells is linked to the cell ability to perform longitudinal directional movement (Petrie et al., 2009). It has therefore been suggested that filopodia and lamellipodia could play a role in the cellular alignment and directional motion observed on unidirectional grooved substrates. Certainly due to their highly dynamic nature, a wide range of filopodia orientations have been reported on grooved substrates, both along or across the grooves. Nevertheless, most studies report that a majority of filopodia grow along the direction of the grooves in aligned cells (Teixeira et al., 2003, 2006; Choi et al., 2007; Fujita et al., 2009; Franco et al., 2011; Kwon et al., 2012; Figure 4B). This observation is nicely illustrated in the study of Teixeira et al. (2006) where corneal epithelial cells perpendicularly oriented on small groove pitch extend filopodia both in the direction of cell orientation but also perpendicularly (i.e., along the grooves). When the pitch was increased cells switched to parallel alignment as well as almost exclusive parallel orientation of filopodia along the grooves (Teixeira et al., 2006). Some studies investigated more carefully the dynamics of filopodia. Fujita et al. (2009) showed that cells on flat or grooved substrates formed filopodia at similar frequency, but that filopodia lifetime was increased on grooves. In addition, filopodia growing perpendicularly to the grooves direction were more rapidly retracted than the ones elongating along the grooves (Fujita et al., 2009; Figure 4C). Mai et al. (2007) observed smooth muscle cell filopodial dynamics in response to the multidirectional cues provided by an orthogonal array of ridges (Figure 1). They showed that cells initially extend protrusions in every directions, but that extensions alongside ridges were quickly retracted, whereas protrusions along the direction of longer ridges were strengthened, resulting in the persistent polarity and migration direction (Mai et al., 2007).

It has been hypothesized that filopodia growth perpendicularly to the grooves will be hindered when facing a ridge/groove wall and therefore retracted, while filopodia can freely adhere, grow and stabilize along the groove. This will generate anisotropic forces that will ultimately orient the cell in the direction of the pattern (Dalton et al., 2001; Teixeira et al., 2003). Accordingly, using aligned or randomly oriented fibers, Kubow et al. (2017) demonstrated that individual protrusions of human fibrosarcoma cells are guided by local fiber orientation, and that the co-alignment of several protrusions on aligned fibers led to the overall polarization of the cell.

More generally, these different studies suggest that the cell is able to generate a global coordination based from the dynamical behavior of multiple protrusions.

#### Topography Sensing in Neurons

##### Growth cone exploration

**FIGURE 5:**
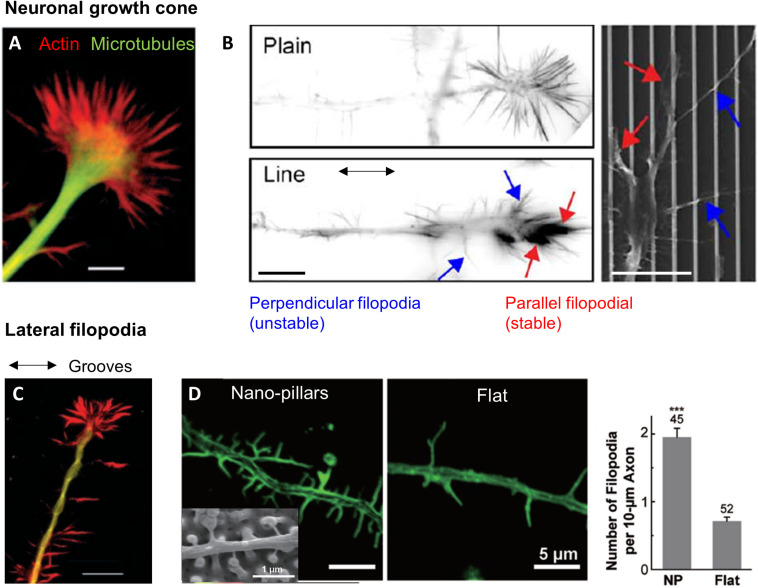
Topography sensing in neuronal cells. **(A)** Structure of a neuronal growth cone (GC). Scale 5 μm. Reproduced with permission from Rajnicek and McCaig (1997). **(B)** Growth cone filopodia on flat or grooved substrate. GC on grooves show two population of filopodia: parallel or perpendicular. Scale 10 μm. From Jang et al. (2010). **(C)** Lateral filopodia along grooves from the neurite shaft on perpendicularly oriented neurites. Scale 10 μm. Reproduced with permission from Rajnicek and McCaig (1997). **(D)** Increased number of lateral filopodia on hippocampal neurites on nano-pillars. Reproduced with permission from Seo et al. (2018). The *** correspond to the *p*-value of the *t*-test in between the 2 groups ***p < 0.001.

Growth cones are the main sensory and protrusive structure of neuronal cells. The growth cone is a hand-like expansion of the growing axon, with a central domain surrounded by an actin-rich lamellipodial region and protruding filopodia (Figure 5A). It is located at the tip of neurites and drives its navigation by sensing and responding to various extracellular signals, such as chemoattractive or repulsive molecules (Tessier-Lavigne and Goodman, 1996). Similarly, it responds to topographical cues present in the extracellular environment of neuronal cells. Growth cone size (higher than the average size of neurites) and mobility determines its area of exploration and therefore likely the scale of topographical variation which can be effectively detected. Accordingly, Li and Folch (2005) showed that the maximal step height that neurites can cross (10 μm) was approximately the size of the growth cone. Growth cone filopodia exploration and adhesion counterbalanced by neurite rigidity and bending could thus be at the basis of neurite depth sensing mechanism, as proposed by Chua et al. (2014). The potential importance of filopodia in neuron contact guidance was further illustrated in a study where the destabilization of filopodia by Cytochalasin D (an actin-polymerization inhibitor) abolished the specific hippocampal neurites responses to different sizes of nanobeads (Kang et al., 2012). Jang et al. (2010) tackled more directly this issue by carefully observing growth cone filopodia on neurites aligning along parallel nanoridges/grooves. They could distinguish two populations of filopodia: thick, actin-rich and stable filopodia aligned in the ridge direction, as well as non-aligned thin and unstable filopodia (Figure 5B). This latter population of filopodia exhibited dynamic instability and rounds of growth and collapse, in contrast to longitudinal filopodia which elongate and stabilize in the direction of the ridges. These results suggest that filopodia are the organelles that allow sensing of the grooved substrate through a stochastic filopodia-mediated search and capture mechanism in neurons (Jang et al., 2010).

In addition, Jang et al. (2010) hypothesized that a crosstalk exists between lateral and longitudinal filopodia in the growth cone. Both types would then contribute to boost neurite growth. Interestingly, Bugnicourt et al. (2014) also suggested (although in a different context, i.e., considering neuron growth on nanopillared instead of grooved surfaces) that the primary signal of accelerated elongation might be given by a population of transverse, weakly adherent filopodia that sense the presence of lateral topographies. Such hypothesis raises again the interesting issue of the integration of local topographical sensing by different subcellular structures toward a global cell response.

##### Role of lateral filopodia on the neuritic shaft

Neurons also often present lateral filopodia along the neuritic shaft whose role in topography sensing remain unclear. On perpendicularly oriented neurites, lateral filopodia extending along the successive parallel ridges were observed at several occasion (Figure 5C), whereas fewer of these filopodia were present on neurites oriented parallel to the grooves (Nagata et al., 1993; Rajnicek and McCaig, 1997; Fozdar et al., 2010). However, destabilization of filopodia by Cytochalasin D did not change the frequency of perpendicularly oriented cells, arguing against a major role of these filopodia in neuronal perpendicular guidance (Rajnicek and McCaig, 1997). Lateral filopodia along the neurite shaft of hippocampal neurons were also observed on arrays of nanoposts where they physically interact with the structures. These lateral filopodia were more numerous on microstructured surfaces compared to flat control, which points toward a role in the response to topography (Seo et al., 2018; Figure 5D). Additional experiments with more specific targeting of this population will be needed to further decipher their role in neuronal contact guidance.

For most cell types, alignment of longitudinal filopodia and protrusions guided by the topography associated with reduced stability of lateral filopodia appears to be a common response to continuous anisotropic topographies and one possible cause of cell alignment and directional movement in these environments.

### Focal Adhesions

**FIGURE 6:**
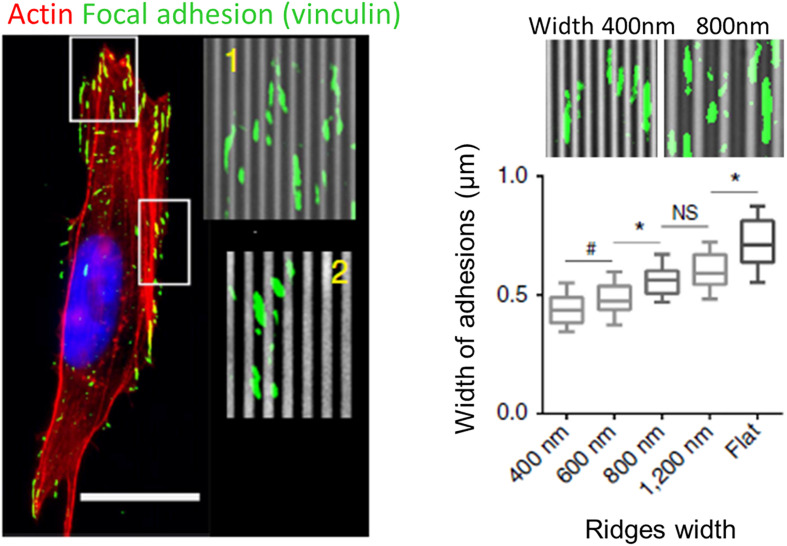
Role of focal adhesions (FA) in topography sensing. Left: cancer cell aligned on a nanogrooved substrate stained for actin (red), vinculin (green), and nucleus (blue). Insets shows aligned FA (1) or non-aligned FA complexes (2). Scale 20 μm. Right: Relationship between ridges width and FA width. From Ray et al. (2017). Symbols correspond to the *p*-values of the statistical tests performed: ^#^*P* < 0.05, *P < 0.001, NS = no significance (ANOVA).

Similar to filopodia, FAs have been proposed to be a primary sensor and actor in contact guidance. A large number of studies using grooved substrates have reported an alignment of FAs in the grating direction, especially for small submicrometric groove/ridge width that encompasses the lateral dimension of FAs (250–500 nm) (Ohara and Buck, 1979; den Braber et al., 1998; Teixeira et al., 2003, 2004; Franco et al., 2011; Saito et al., 2014; Azatov et al., 2017; Ray et al., 2017; Figure 6). More precisely, FAs were seen to assemble and elongate either preferentially on ridges (Ohara and Buck, 1979; den Braber et al., 1998; Ferrari et al., 2010, 2011; Franco et al., 2011; Azatov et al., 2017) or on both ridges and grooves (Ray et al., 2017; Tabdanov et al., 2018). In addition, scaling of FAs width with the size of the ridges have been reported (Teixeira et al., 2003; Saito et al., 2014; Ray et al., 2017; Figure 6). When the lateral dimensions of grooves increase (above 1–2 μm), confinement on FAs growth decreases and FAs can adopt more oblique directions (den Braber et al., 1998). Conversely, when groove/ridges width are small enough, FAs can bridge multiple ridges. On grooves pitch ranging from 400 nm to 4 μm, Teixeira et al. (2006) accordingly described an optimal range of lateral dimension for FAs alignment (800–1200 nm), under and above which oblique FAs (over multiple or single ridge, respectively) were also observed. A similar result was observed on neuronal PC12 cells. For the smallest ridge width (500 and 750 nm), FAs were visibly aligned with the nanograting. With increasing ridge width (up to 2 μm), the number and size of misaligned FAs increased, correlated with gradual loss of whole cell alignment and elongation (Ferrari et al., 2011).

These observations led to the FA restriction theory, first proposed by Ohara and Buck (1979), stating that continuous unidirectional cues provide an essentially one-dimensional substrate upon which adhesions can only elongate and mature in one direction. Because adhesions grow linearly, those elongating in the direction of ridges have a large area on which to grow, while those elongating perpendicularly are restricted to the width of the ridge, limiting their growth and maturation.

A first possible mechanism by which this angular modulation of FAs growth by topography can affect the whole cell response is via cell protrusions/filopodia. Because cell protrusions establish adhesions with the substrate, adhesion elongation along groove direction will guide protrusions in the same direction, thereby promoting alignment and oriented movement. This was illustrated by the work of Fujita et al. (2009), who observed that MSC protrusions extending in the direction of nanogrooves were stabilized and contained elongated mature FAs. Conversely, in cell protrusions extending perpendicularly to the grooves, FAs align parallel to the groove direction, but are fragmented, which leads to unstable and easily retracted protrusions (Fujita et al., 2009). This observation was also made on PC12 neuron-like cells with growing neurites aligned with nanogrooves (250 nm depth and 500 nm pitch). After addition of nerve growth factor (NGF) triggering PC12 cells differentiation, bright, long and stable FAs were seen elongating on ridges at the tip of aligned neurite-like processes. In contrast, interaction with the nanograting at the tip of misaligned processes hinders the increase of the length of FAs, thus blocking their maturation (Ferrari et al., 2010). A second alternative or complementary mechanism is that FAs can affect the whole cell orientation via their link to the cytoskeleton, as we will detail in a coming section.

### Cell-Topography Interface and Topography Sensing by Membrane Curvature

As the interface between the cell and the extracellular environment, the whole cell membrane is a privileged sensor of substrate topography. The topic of the membrane-topography interface has mainly been explored in the context of high aspect ratio (1–200) nanostructures, which we are going to focus on in this section. For more details on these topics, the reader is referred to dedicated recent reviews (Lou et al., 2018; Higgins et al., 2020).

Cell membrane has been shown to tightly conform to vertical topographical cues. Membrane deformation is dependent on the size and spacing of the structures: a study of Bianxiao Cui’s team shows that the cell body of cortical neurons rests atop of closely spaced (1 μm), 1 μm high nanopillars of large (>300 nm) diameter, while with increased spacing the cell is able to engulf nanopillars up to 500 nm in diameter. Alternatively, when the spacing between nanopillars is kept at 1 μm, the transition between suspension and engulfment can be observed as the diameter decreases from 500 to 200 nm (Figure 4D; Hanson et al., 2012). Many other studies have shown using electron microscopy that cell membrane can engulf high aspect ratio vertical nanostructures, with no clear evidence of membrane rupture (Lin et al., 2014; Santoro et al., 2017; Dipalo et al., 2018; Gopal et al., 2019).

The development of FIB-SEM (a combination of focused ion beam FIB and scanning electron microscopy SEM) which enables the visualization of a cross-section of the cell-material interface at high resolution was instrumental in these studies. In particular, this technique allows to measure the distance between the cell membrane and the material surface, which decreases from around 100 nm on flat surface to 15 nm for nanopillared substrates (diameter 200–1500 nm, height 1 μm, spacing 3–5 μm) (Santoro et al., 2017). Interestingly, this distance was increased to 400 nm on the inverse topography of invaginating structures (nanopores) around which the cell membrane hardly deformed (Figure 4E; Santoro et al., 2017). These differences suggest that the plasma membrane interacts with protruding and invaginating surface topologies in fundamentally different ways.

The high membrane deformation observed on these high aspect ratio substrates has led to the “curvature hypothesis” stating that membrane deformation can drive the cell response to topography via the accumulation/recruitment of proteins and subsequent activation of signaling pathways (Lou et al., 2018). Different studies reported both enhanced and more dynamic endocytosis in cells interacting with nanopillars, respectively assessed by an increased amount of endocytosed dextran (Teo et al., 2011) and decreased puncta lifetime of the endocytosis-related protein clathrin (Zhao et al., 2017). In addition, high membrane curvature generated at the site of nanopillars induced the local accumulation of clathrin and its partner protein dynamin-2 (Zhao et al., 2017). Similarly, Galic et al. (2012) used alternating stripes of nanopillars and flat surfaces to show within a single cell a preferential accumulation in the nanopillars regions of the N-BAR domain proteins amphiphysin 1 (a regulator of endocytosis) and nadrin 2 (a regulator of actin polymerization). Overall, this experimental evidence suggests that nanostructures enhance endocytosis by recruiting curvature-sensitive proteins.

In addition, various studies have reported a local accumulation of F-actin at the location of nanopillars (Bonde et al., 2013; Beckwith et al., 2015; Zhao et al., 2017; Hansel et al., 2019; Lou et al., 2019). The mechanisms associated with this local actin polymerization remains unclear. Actin accumulation at the nanopillars does not seem to be associated with FAs or stress fibers (Bonde et al., 2013; Lou et al., 2019) and does not require acto-myosin contractility (Hansel et al., 2019). However, this actin polymerization is membrane curvature dependent, since it was observed to be stronger for smaller diameter nanopillars, where membrane curvature is higher (Lou et al., 2019).

In conclusion, topography-induced membrane curvature appears like a key mechanism underlying cells recognition and response to surface topography. However, whether this mechanism is universal and applies for a wide range of topographical cues geometries (beside high aspect ratio structures) remains to be elucidated.

## Key Molecular Players Involved in Contact Guidance

### The Cytoskeleton and Associated Partners

Cellular shapes, orientation, and motility are intimately determined by the arrangement and the dynamics of cytoskeletal elements: actin, microtubules, and intermediate filaments. The observation of the cytoskeleton in the context of contact guidance was initiated in early studies (Oakley and Brunette, 1993; Wojciak-Stothard et al., 1995), and they now constitute key elements toward the understanding of the molecular mechanisms involved in this process.

Due to the lack of converging results and a still relatively less abundant literature regarding the role of intermediate filaments in contact guidance, we will only review in this section the literature on the most studied filaments, i.e., microtubules and actin. This lack of literature on the role of intermediate filament might be due to the great diversity of this cytoskeletal family. A couple of studies interested in different sub-families of intermediate filaments can nevertheless be mentioned. For instance, vimentin intermediate filaments were shown to display appreciable alignment in adult meningeal cells (Manwaring et al., 2004). Glial fibrillary acidic protein (GFAP) intermediate filaments were also observed to orient to some extent in the direction of grooves in astrocytes (Singh et al., 2015). Holle et al. (2017) addressed the influence of keratin filaments in pancreatic cancer cells and showed that although keratin filaments did not align substantially in the direction of grooves, the disruption of the keratin network changed the response of cells to grooves and pillared substrates.

#### Actin Related Players

##### Alignment of the actin network

**FIGURE 7:**
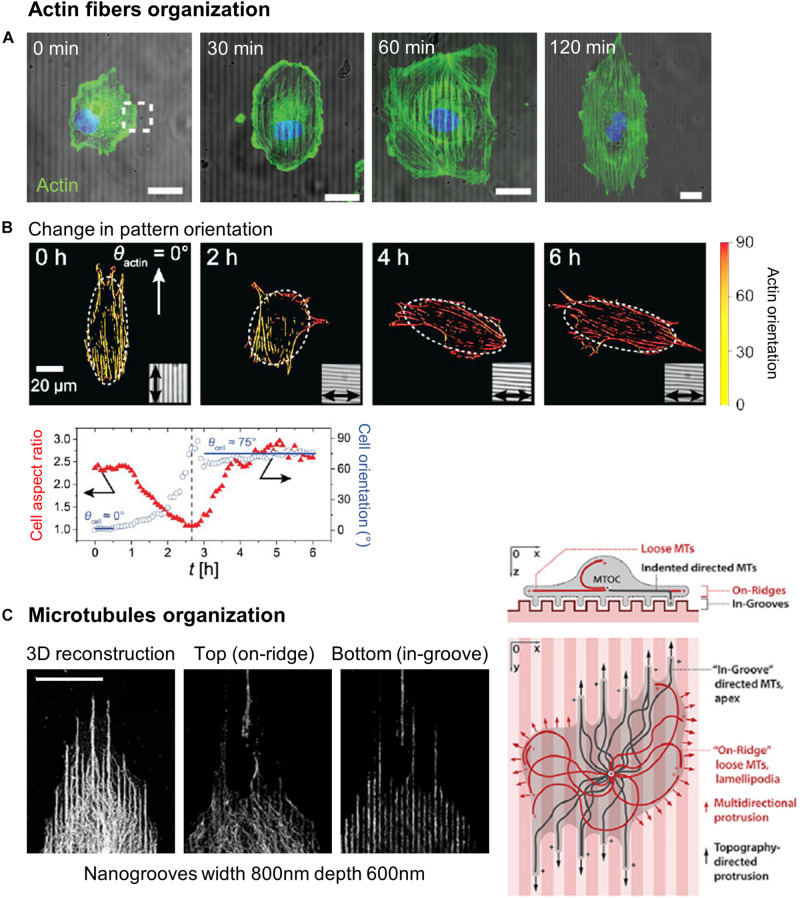
Cytoskeleton organization on microstructured substrates. **(A)** Actin staining of endothelial cells on 2 μm wide, 350 nm deep grooves at different time points showing actin fibers alignment shortly after cell orientation along the grooves. Scale bars 20 μm. From Sales et al. (2017) published by the Royal Society of Chemistry. **(B)** C2C12 myoblasts transfected by LifeAct-GFP on microwrinkles substrates. After 2 h, the orientation of the wrinkles is changed by 90°. Actin orientation closely follows cell reorientation quantified by the cell orientation (red) and aspect ratio (red). Reprinted with permission from Linke et al. (2019). Copyright 2016 American Chemical Society. **(C)** Microtubules network organization in cancer cells on grooves reveals a loose “on ridge” network and a “in groove” directed network. Scale 20 μm. From Tabdanov et al. (2018).

Alignment of actin filaments and stress fibers has been widely observed in cells orienting in grooved substrates (Wojciak-Stothard et al., 1995; Walboomers et al., 2000; Teixeira et al., 2004, 2006; Biggs et al., 2008; Kim et al., 2009a; Franco et al., 2011; Azatov et al., 2017; Sales et al., 2017; Figures 7A,B). More recently, this observation was completed by dynamic studies of the actin network. Linke et al. (2019) studied the dynamics of mouse myoblast morphological response and actin organization on wrinkled substrates. During the initial spreading, they measured a clear correlation between cell orientation and elongation parallel to the wrinkles and the orientation of actin fibers, suggesting that shape adaptation to topography is guided by the actin cytoskeleton. This result was reinforced by dynamically changing the orientation of the wrinkles by 90° by application of axial strain. In this situation, the adaptation of cell shape with the new pattern direction (in a few hours) was synchronized with the reordering of the actin network (Figure 7B; Linke et al., 2019). Actin stress fibers dynamics was also analyzed in more details in cancer associated fibroblasts (expressing EGFP-palladin, which preferentially labels actin stress fibers) on parallel arrays of nanoridges. Tracking of actin stress fibers revealed enhanced dynamics on nanostructures, with preferred movement along the ridges (Azatov et al., 2017).

##### Acto-myosin contractility and associated signaling

A number of studies observed reduced contact guidance following acto-myosin pharmacological perturbations, pointing toward a role of acto-myosin driven contractility in topographical response (Wojciak-Stothard et al., 1995; Gerecht et al., 2007; Mai et al., 2007; Saito et al., 2014; Lee et al., 2016). However, some apparent discrepancies still persist in the literature on the effect of acto-myosin inhibition and its role on contact guidance (Table 4). For instance, some studies report that the application of acto-myosin perturbing agents have no or only slight effects, especially at the initial stages of contact guidance (Oakley and Brunette, 1993; Walboomers et al., 2000; Franco et al., 2011; Sales et al., 2017). These observations led to the hypothesis that actin polymerization, but not actomyosin contraction, drives initial contact guidance events. This early contact guidance might be a “passive” process, requiring only rather simple cellular machinery like actin polymerization. In contrast, later stages of contact guidance appear more sensitive to loss of actomyosin contractility suggesting a more “active” process requiring contractility and mechanotransduction to further adapt to the substrate. This model relates to the “Curtis and Clark” theory (1990) stating that cells react to topography primarily at lines of discontinuity in the substratum by actin nucleation, and supported by other studies which observed F-actin condensations at the edge of grooves/ridges rapidly after cell seeding (Oakley and Brunette, 1993; Wojciak-Stothard et al., 1995).

**TABLE 4:** Cytoskeleton organization and effect of cytoskeleton inhibitors on contact guidance.

Cell type	Type of topography	Dimensions	Cytoskeleton organization	Drugs/KO	Cell response	References
*Dictyostelium discoideum*	Grooves	Depth ∼600 nm Width 0.4–10 μm Spacing 250 nm	Preferential actin polymerization waves along nanoridges	Latrunculin	Disrupted CG	Driscoll et al., 2014
				Myosin II KO	Undisrupted CG	
T cells	Grooves	Depth 500 nm Width 700 nm Spacing 350 nm		Latrunculin A 12.5 nM	Disrupted GC, decreased motility	Kwon et al., 2012
				Blebbistatin 50 μM	Undisrupted CG	
Smooth muscle cells	Grid of orthogonal ridges	Depth 2 μm Width 2 μm Spacing 20 μm × 9–50 μm	Focal adhesions (FA) along the ridges	Blebbistatin 50 μM	Disrupted GC	Mai et al., 2007
				Y27632 20 μm	Disrupted GC	
				ML-7 20 μM	Undisrupted CG	
MDA-MB-231 (breast carcinoma, mesenchymal)	Grooves	Depth 600 nm Width and spacing 800 nm	Stress fibers (SF) alignment FA alignment, width proportional to ridge width	Blebbistatin 50 μM	Disrupted CG	Ray et al., 2017
Human embryonic stem cells	Grooves	Depth, width, spacing 600 nm	Cytoskeleton alignment	Actin disrupting agents (Cytochalasin D 1 mg/mL, Latrunculin B 8 μM)	Disrupted CG	Gerecht et al., 2007
Fibrosarcoma cells (HT-1080)	Fibers	Diameter 700 nm	Long and stable FA along the fibers, small and unstable FA ⊥	Blebbistatin 50/100 μm	Undisrupted CG	Kubow et al., 2017
				Y27632 20 μM + ML7 10 μM	Undisrupted CG although decreased FA lifetime	
Smooth muscle cells	Grooves (shape of wrinkles)		Small, aligned FA	Blebbistatin 5 μM	Disrupted CG	Saito et al., 2014
Retinal pigment epithelial cells (RPE-1)	Grooves	Depth 0.35/1 μm Width 1 μm Pitch 2 μm	SF, FA and microtubules (MT) alignment	Cytochalasin D 1/2 μM	Disrupted CG except for deep gratings	Lee et al., 2016
				Nocodazole 1 μM	Disrupted CG on deep gratings	
Fibroblasts	Grooves	Depth 0.5, 1, 2, 5 μm Width 5, 10, 25 μm	SF and FA alignment, followed by MTs	Cytochalasin D 1 μg/mL	Reduced orientation and elongation	Wojciak-Stothard et al., 1995
				Colcemid 2 μg/mL	Reduced orientation and elongation	
				Taxol 12 μM	Reduced elongation but not orientation	
Endothelial cells	Grooves	Depth 0.1–2 μm Width and spacing 1/5 μm	SF and FA alignment	Blebbistatin 50 μM	Undisrupted spreading but disrupted CG	Franco et al., 2011
				Y27632 10 μM		
Endothelial cells	Grooves	Depth 200, 350, 650 nm Width 2, 10 μm	SF alignment after 30 min	Blebbistatin 20 μM	Altered alignment but not elongation	Sales et al., 2017
Fibroblasts	Grooves	Depth 0.5 μm Width 1–10 μm	SF aligned after 4 h	Cytochalasin B 2.5 μmol/L	Delayed spreading but undisrupted CG	Walboomers et al., 2000

Contractility generated by acto-myosin is under the control of a complex signaling network, dominated by the Rho-associated kinase ROCK which increases the activity of myosin II by phosphorylation of the myosin light chain (MLC). Overall, inhibition of ROCK by the chemical agent Y27632 therefore leads to impaired contact guidance, similar to the effect of myosin II inhibition (Mai et al., 2007; Franco et al., 2011).

ROCK and myosin II contractility are involved in stabilizing cell polarity by spatially coordinating protrusive activity (Lo et al., 2004). Accordingly, ROCK-inhibited fibroblasts on microprinted lines of fibronectin present multiple protrusive fronts and are unable to orient their protrusions along the lines (Ramirez-San Juan et al., 2017). Similarly, Mai et al. (2007) observed that smooth muscle cells treated with Y27632 can send protrusions along the ridges of a grid pattern but, contrary to control cells, are not able to coordinate protruding activity and to down regulate protrusions in minor directions in order to generate a directional movement along the main axis of the rectangular array. More generally, all signaling involved in the control of cell protrusion are likely to influence cell response to topography. For instance, the Arp2/3 complex is responsible for actin branching and is an essential component of lamellipodial protrusions at the leading edge (Wu et al., 2012). Inhibition of Arp2/3 with the inhibitor CK-666 has been shown to increase contact guidance and cell elongation on fibronectin lines by reducing protrusion size (Ramirez-San Juan et al., 2017).

In addition, Rho/ROCK-mediated contractility exerted by actin stress fibers on FAs plays an important role in FA maturation, activation of downstream signaling (Burridge and Guilluy, 2016; Warner et al., 2019) and the overall reinforcement of contact guidance as will be detailed below.

##### Focal adhesions and associated signaling

Actin stress fibers originate at FAs, which themselves orient along topographies (see section “Focal Adhesions”). The direction of FAs might thus guide the direction of the stress fibers, explaining their overall alignment, and the ultimate change in cell shape and orientation. This model of contact guidance mechanism was elegantly demonstrated by Ray et al. (2017). They showed using different cancer cell lines that physical confinement of FAs by nanogratings generates anisotropic traction forces leading to directional cell orientation and migration. Accordingly, relaxing FAs confinement by reducing FA size with the drug rotenone, or reducing myosin-regulated contractile force with blebbistatin led to decreased cell alignment. Disturbing the link between FAs and actin stress fibers while preserving FA morphology (with β1-integrin function-blocking antibody) led to the same phenotype, showing overall that contact guidance involves some substrate driven force generation (Ray et al., 2017).

Aside from its structural role, the formation of FAs induces several intracellular signaling processes. Among the multiple signaling molecules that are recruited to FAs upon integrin clustering are non-receptor protein-tyrosine kinases such as FAK (focal adhesion kinase). As the main transducer of extracellular physical cues into intracellular signals, FAs and its associated downstream signaling have been naturally investigated in the context of contact guidance. In endothelial cells interacting with micrometric grooves, activated FAK (phospho-FAK) signal is increased by 80% compared to cells spreading on a flat surface (Franco et al., 2011) and on pillared surfaces FAK−/− fibroblasts show no detectable response to the topography (Frey et al., 2006), suggesting a role of FAK in this process. On the contrary, Dreier et al. (2012) observed an increased percentage of aligned human corneal epithelial cells along grooves (pitch 400 nm to 4 μm) when FAK was knock-down by siRNA. However, the authors also noted an increased expression of Nesprins (linking the nucleus to the cytoskeleton) when FAK was silenced and hypothesized that Nesprins upregulation could compensate for FAK loss and allow cell response to topographical cues. Similarly, downregulating FAK by siRNA in hMSC had only a limited effect on cell morphology and alignment to nanogratings, but impaired the topography-induced neuronal hMSC differentiation (Teo et al., 2013). These results highlight the complex and yet central role of the signaling network involved in cell response to topography downstream of FAs, which can also depend on the cell type or type of topographies used.

#### Microtubule Network

In contrast to the acto-myosin network, the role of microtubules in contact guidance has received less attention. Similar to actin filaments, alignment of microtubules have been reported in aligned cells on grooved substrates (Oakley and Brunette, 1993; Biggs et al., 2008; Lee et al., 2016). Oakley and Brunette (1993) even observed that microtubules were the first element to align to the topography, even before actin filaments, pointing toward a potentially important role of microtubules in topography response.

Lee et al. (2016) showed on retinal pigment epithelium-1 (RPE-1) cells that microtubules alignment was more pronounced on 1 μm-deep grooves than on 0.35 μm-deep grooves and that microtubules (but not actin) disruption impaired contact guidance on the deeper grooves (Lee et al., 2016). This indicates that microtubules are influenced by geometrical constraints, and that they could provide a complementary or compensatory role in contact guidance in addition to actomyosin. While investigating the response of fibroblasts and epithelial cells to intersecting grooves, it was also observed that microtubules adapted more closely to the corner of the intersection than F-actin bundles. Inhibition of microtubules nucleation by colcemid attenuated cell conformation to the intersection of the grooves. The authors therefore hypothesized that microtubules could determine cell orientation in response to conflicting topographic cues (Hamilton et al., 2009).

More recently, Tabdanov et al. (2018) performed an in-depth analysis of the microtubules in two cancer cell lines (MDA-MB-231 and MIA-Paca-2). They distinguished two different microtubules network: a strongly aligned “in groove” network on the ventral side of the cell (associated with aligned actin stress fibers), and a more isotropic network on the dorsal side (associated with actin transverse arcs) (Tabdanov et al., 2018; Figure 7C). The authors hypothesized that this first microtubule network is sterically trapped inside the nanogrooves, providing a structural regulation of cell protrusion orientation and stability and enhancing overall cell elongation along topographical cues. Accordingly, loss of dynamic in-groove microtubules by nocodazole treatment results in a more isotropic mode of protrusion, where cells are not as robustly directed by the nanostructures. Moreover, microtubule localization to nanogrooves appears also regulated by the balance between actin stress fibers and transverse arcs associated with more directed versus multidirectional protrusions, respectively.

Finally, microtubules appear structurally and mechanically involved in the process of contact guidance. Further studies will aim at specifying this role and the network of interaction with other intracellular elements involved in contact guidance, such as actin. It would be particularly interesting to address these questions in neuronal cells, which functions usually heavily rely on the microtubule network.

#### Summary: General Model of Contact Guidance by Anisotropic Cues

To conclude, contact guidance appears mediated by a complex cross-talk between FA maturation, directed protrusions and cytoskeletal activity. The confined growth of FA leading to oriented acto-myosin network appears today like the most general and accepted model of cellular alignment by continuous unidirectional topography. However, this model still lacks the contribution of other cytoskeletal networks such as microtubules or intermediate filaments. In addition, this conclusion should be balanced and modulated by the cell type specific determinants and intracellular organization. Indeed, cells rely differently on the various elements of its intracellular machinery. This can explain cell type specific responses to topography. For instance, contact guided migration by nanogrooves of the amoebae *Dictyostelium discoideum*, which is devoid of FA appears to be mediated by actin polymerization waves along nanoridges, independently of myosin II (Driscoll et al., 2014). This holds also true for neurons, which exhibit few and less organized FAs compared to most cells, and still exhibit contact guidance. This result suggests that multiple non-exclusive mechanisms of contact guidance might coexist for different cell types, or for different types of topographical environments. Following this idea, Ray et al. (2017) proposed based on experimental results a model of cell response to topography based on the relative weight of the different intracellular modules such as FA size or F-actin morphology. In this model, cells with large FAs, highly constrained FA growth, strong stress fibers and high traction anisotropy (typical mesenchymal cell) are predicted to exhibit strong contact guidance while cells with small FAs, diffuse F-actin and lower traction anisotropy (amoeboid cells) would show a milder response.

### Intracellular Signaling Associated With Contact Guidance

The signaling pathways involved in topography response downstream of cytoskeleton-associated proteins are still not fully identified. It is tempting to postulate that pathways involved in mechanotransduction might be at least partly involved in topography-associated responses. As such, YAP-TAZ signaling is a promising player.

#### YAP-TAZ Signaling

The role of Yes-associated protein (YAP) transcription factor coupled with its transcriptional coactivator with PDZ-binding motif (TAZ) in the process of mechanotransduction (i.e., the transduction of external mechanical stimuli into change in gene expression) has recently been emphasized (Totaro et al., 2018). Schematically, different signals such as ECM stiffness or contractility can activate cytoplasmic YAP/TAZ, which is transported to the nucleus where it can induce the expression of target genes. However, the effect of topography on the activation and intracellular localization of YAP is still largely unknown. YAP/TAZ expression and localization was investigated in corneal epithelial cells cultured on grooves (pitch 400 nm to 4 μm, depth 300 nm). While mRNA expression of YAP and TAZ increased in these cells, no difference in spatial localization was observed compared to control cells on planar surface. No change in cell alignment was visible after treatment with siRNA against YAP, but siRNA against TAZ led to an increased alignment (Raghunathan et al., 2014). More recently, Tonazzini et al. (2020) similarly reported only a slight increase in YAP nuclear/cytoplasmic ratio in PC12 cells on nanogrooves, and no change in YAP expression levels (Tonazzini et al., 2020). Overall, these two studies suggest that YAP/TAZ pathway is only partially involved in contact guidance, at least in the response to grooves for these cell types. We can nevertheless mention that several studies interested in the topography-induced differentiation of stem cells reported an increased YAP cytoplasmic localization of stem cells cultured on various types of topographies (Song et al., 2016; Chen et al., 2018; Loye et al., 2018). However, this response might not be directly linked to the detection of topography but rather to the differentiation pathway. Strengthening this hypothesis, Ankam et al. (2015) observed a decrease in YAP expression when human embryonic stem cells differentiate toward the neuronal lineage, but no difference in overall expression and localization of YAP between the cells cultured on nanogratings and unpatterned substrates, suggesting that YAP might not be required for the topography-induced differentiation of these cells. Further studies will therefore be needed to elucidate the role of YAP/TAZ in contact guidance.

## Conclusion and Future Directions

The literature now provides extensive evidence for the phenomenon of contact guidance in many cell types, in interaction with a large panel of microfabricated topographies. We have first tried in this review, without claiming to be exhaustive, to provide a phenomenological overview of cellular responses organized by categories of topographies. To go beyond phenomenology, we have then dedicated a second part of this review on the structures supporting contact guidance at the subcellular (filopodia, FAs or cell membrane) down to the molecular (cytoskeletal networks) levels.

However, multiple questions and challenges still lie ahead. The next step in the understanding of the mechanisms involved in contact guidance is to reach the molecular level and decipher the signaling pathway involved. Some studies mentioned in the last part of this review have started to tackle this issue and identify some key molecular players. However, we still lack a complete and coherent picture of the network of pathways involved. *In vivo*, cells detect a wide range of physical cues in their environment, including mechanical properties such as substrate stiffness. How much similarity exists between the subcellular and molecular mechanisms underlying topography and stiffness detection is still unclear and should drive future research.

Technical improvements will surely drive the next wave of contact guidance investigations. These improvements include advances in fabrication techniques (Cho et al., 2019; Eltom et al., 2019) (further miniaturization, control of substrate geometry and surface properties, development of 3D scaffolds, biocompatible, and stretchable devices for biomedical applications, etc.), advances in microscopy for a better visualization of cell-topography interaction (super-resolution microscopy, FIB-SEM) and advances in analysis methods (multi-omics approaches for instance).

Beyond its fundamental interest, the phenomenon of contact guidance has been recognized as key to many potential biomedical applications. For instance, the role of the topography in stem cell differentiation is a rapidly emerging research line, providing insights in both the fundamental mechanisms of cell differentiation and its application to regenerative medicine (Ravichandran et al., 2009; Cosson et al., 2015). The design of implants is another scope for the implementation of micro-engineered surfaces in biomedical sciences. In the particular case of cardio-vascular pathologies, grooved-surface implants are developed to guide migration and improve re-endothelization (Lutter et al., 2015; Tan et al., 2017). The field of neuroregenerative medicine has also benefited from advances in contact guidance research, and the development of bioengineered scaffolds is now explored as a therapeutic solution to improve neurite outgrowth in the case of nerve injury, in particular of the peripheral nervous system (Straley et al., 2009; Shea and Mok, 2018).

More generally, the integration of contact guidance in a more physiological landscape including other environmental cues (e.g., shear stress mimicking the blood circulation) is now key for both biomedical applications and the fundamental understanding of the combinatorial response of cells to various environmental cues. Micro-engineered environments are particularly suited to explore this issue, as already demonstrated by some works exploring the joined influence of the topography and shear stress (Morgan et al., 2012), or topography and chemical cues: growth factors (Gomez et al., 2007a), adhesive molecules (Charest et al., 2006) or chemoattractants (Kundu et al., 2013). Such work should be developed in the future and will be instrumental in the development of efficient synthetic biomedical devices.

Overall, the study of contact guidance is at a turning point of its evolution. After a feverishly active period of exploration of the cellular responses to the topography stimulated by the almost unlimited possibilities of developing micro-topographies, the field must now progress in two opposite directions. The first one would be to understand the fundamental mechanisms of topographical sensing by taking advantage of the minimalist approach allowed by micro-engineered technologies. The second and opposite effort should be made on the integration of contact guidance into a more complex, physiological context, leading ultimately toward clinical applications. The seminal observation of R.G. Harrison and P. Weiss at the beginning of the 20th century has thus led to a productive, and still highly active research field at the crossroads among the fields of fundamental biology, biophysics, material sciences, and clinics.

## Author Contributions

CL and CV wrote the review. Both authors contributed to the article and approved the submitted version.

## Conflict of Interest

The authors declare that the research was conducted in the absence of any commercial or financial relationships that could be construed as a potential conflict of interest.
